# Towards homochiral supramolecular entities from achiral molecules by vortex mixing-accompanied self-assembly†
†Electronic supplementary information (ESI) available. See DOI: 10.1039/c8sc04687e


**DOI:** 10.1039/c8sc04687e

**Published:** 2019-01-07

**Authors:** Yutao Sang, Dong Yang, Pengfei Duan, Minghua Liu

**Affiliations:** a Beijing National Laboratory for Molecular Science , CAS Key Laboratory of Colloid, Interface and Chemical Thermodynamics , Institute of Chemistry , Chinese Academy of Sciences , Beijing 100190 , China . Email: liumh@iccas.ac.cn; b CAS Center for Excellence in Nanoscience , Division of Nanophotonic , CAS Key Laboratory of Nanosystem and Hierarchical Fabrication , National Center for Nanoscience and Technology (NCNST) , Beijing 100190 , China . Email: duanpf@nanoctr.cn; c University of Chinese Academy of Sciences , Beijing 100049 , China; d Collaborative Innovation Centre of Chemical Science and Engineering , Tianjin 300072 , China

## Abstract

Achieving homochirality is challenging both at the molecular and the supramolecular levels. While the origin of molecular homochirality still remains mysterious, the fabrication of homochiral assemblies from achiral molecules has attracted considerable interest since it provides many clues to understand the origin of molecular chirality. Here, by using a vortex mixing-accompanied self-assembly strategy, we obtained near-unity homochiral entities with controlled handedness from supramolecular gels that consist of exclusively achiral molecules without any chiral additives. The common supramolecular gelation process *via* heating and cooling of the achiral molecules only resulted in racemic gels. However, if vortex mixing is applied during the self-assembly, near-unity homochiral assemblies with uncontrolled handedness were obtained. Vortex mixing during the nucleation stage was found to be crucial in this case. On the other hand, if a small amount of the above vortex mixing produced assemblies was added as chiral seeds into the racemic gels, the racemic gels turned into near-unity homochiral suspensions with controlled handedness *via* a ripening process. Our studies provide an intriguing approach for achieving homochiral supramolecular assemblies from achiral molecules.

## Abstract 2

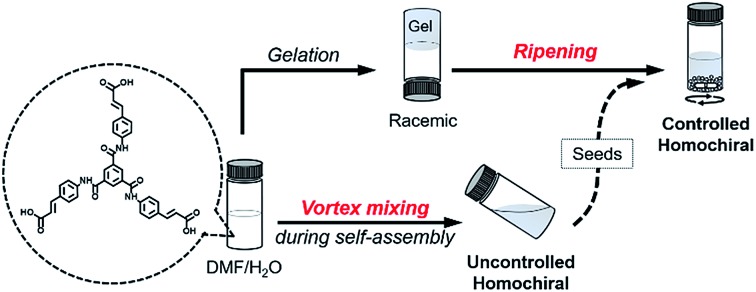
By using a vortex mixing-accompanied self-assembly strategy, homochiral entities with controlled handedness were obtained from exclusively achiral molecules.

## Introduction

Homochirality in biological systems, wherein almost all of the amino acids and sugars found in living organisms are *L*- and *D*-enantiomers, respectively, is one of the most mysterious phenomena found in life.^1^,^2^ The origin of such molecular homochirality still remains unknown. On the other hand, chirality can be observed at various scales and it is a big challenge to obtain the homochiral supramolecular assemblies beyond the molecule. The latter case is particularly interesting since the homochiral assemblies from achiral molecules can provide many clues to understand the origin of molecular chirality.^3^,^4^

So far, homochirality in self-assembly systems has been achieved by only a few methods, including total spontaneous resolution and Viedma ripening.^5^,^6^ The latter case has been proved to be more reliable in reaching an enantiopure product because the nucleation of undesired enantiomers still occurred during spontaneous resolution.^7^ However, these above strategies for homochiral assemblies are limited in solid systems with crystals.^8^,^9^ The reason is that the chiral discrimination is easier to realize in the solid state, particularly for the molecules which have a better affinity for the same enantiomer (to form racemic conglomerate crystals) than for the opposite enantiomer (to form a racemic compound or true racemate) during crystallization. Unfortunately, only small amounts of molecules meet the prerequisite of conglomerate crystallization.^9^,^10^

Different from the crystals, the supramolecular system is usually a dynamic system, where assembly and disassembly processes occurred simultaneously. In order to realize homochirality, the stability of the preformed nanostructures is very important. For example, homochirality is difficult to achieve in diluted solution due to the weak stability and the fast exchange between the molecules and assemblies.^11^ However, supramolecular gel lies in between the solid (or crystals) and diluted dispersion. It might be possible to realize homochirality.

Recently, many achiral molecules have been reported to form chiral nanostructures and exhibited macroscopic chirality *via* spontaneous symmetry breaking, such as amphiphile assemblies,^12^,^13^ liquid crystals,^14^–^16^ dye aggregates,^17^,^18^ Langmuir or Langmuir–Blodgett (LB) films,^19^–^21^ two-dimensional (2D) assemblies,^22^–^24^ supramolecular gels,^25^–^29^ and other systems.^5^,^30^ However, only preferential chiral nanostructures (one enantiomer is more abundant than the other) rather than homochiral assemblies (only one enantiomer) were obtained. In addition, the selection of the chirality is still quite random in most cases. Several methods like vortex motion generated by rotary evaporation or magnetic stirrers and other external influences like a magnetic field and circularly polarized light (CPL) have been applied to select and amplify the chirality of these achiral self-assembly systems.^31^–^37^ However, obtaining homochiral supramolecular assemblies fabricated from achiral molecules is still very challenging, let alone controlling their chirality without any chiral additives.

**Fig. 1:**
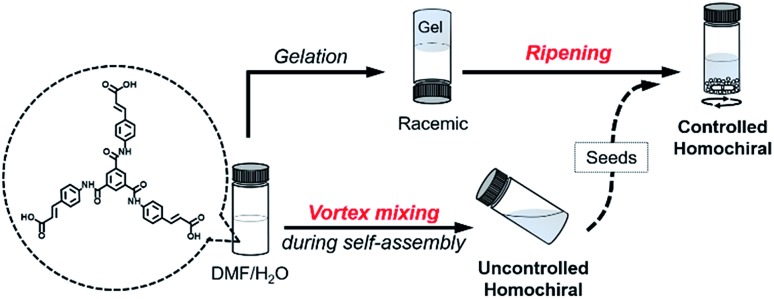
Schematic illustration of this work. Achiral *C*_3_-symmetric monomers can form supramolecular gels in DMF/H_2_O. The common gelation process only results in racemic gels. If a vortex mixing is applied continuously during self-assembly, near unity homochiral assemblies (suspension state) with randomly distributed handedness are obtained (uncontrolled). The obtained homochiral assemblies can be used as chiral seeds and convert the racemic gels into controlled homochiral suspensions *via* a ripening process.

Here, we present a vortex mixing-accompanied self-assembly strategy for fabricating near-unity homochiral assemblies from exclusively achiral molecules without any chiral additives, as illustrated in Fig. 1. The achiral monomers can form racemic supramolecular gels through the common gelation process. Since gel formation is thermally reversible, the assembly and disassembly can be easily manipulated by heating and cooling. It was found that if vortex mixing treatment is applied continuously during the self-assembly process, we obtain near-unity homochiral assemblies (only one enantiomeric assembly) but without selection of the chirality. In this case, vortex mixing during the nucleation stage was found to be crucial. In addition, by using a small amount of assemblies obtained *via* the above vortex mixing as chiral seeds, a subsequent ripening process leads to the conversion of the racemic gels to the identified homochiral state with the seeds (Fig. 1). This operation refers to the process that transfers the already formed supramolecular assemblies from racemic to near-unity homochiral assemblies without the initial nucleation stage. Because we can know the handedness of the seeds *via* circular dichroism (CD) measurement, we can obtain both chirality controlled and near-unity homochiral assemblies without any chiral additives.

## Results and discussion

### Chiral nucleation and amplification through vortex mixing

**Fig. 2:**
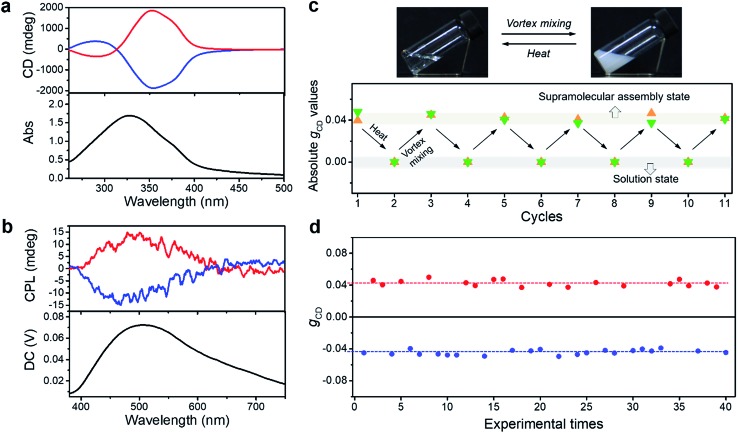
Characterization of the supramolecular assemblies formed through vortex mixing. (a) CD and UV-vis absorbance spectra of the BTACA assemblies. (b) CPL spectra of the BTACA assemblies excited at 320 nm. (c) Schematic representation of thermally reversible supramolecular assembly (top) and the absolute *g*_CD_ values of two independent samples (green and orange triangles) as a function of the number of heat-vortex mixing cycles (bottom). (d) Statistical distribution of *g*_CD_ values from 40 BTACA samples obtained through vortex mixing. BTACA concentration: 5.54 mM in DMF/H_2_O (1 : 1 v/v).

The achiral molecule we used is a derivative of benzene-1,3,5-tricarboxamides (BTAs),^38^ in which three peripheral cinnamic acid moieties are covalently connected to the core benzene ring through the amide bond (BTACA), as shown in Fig. 1. BTACA is insoluble in most common solvents except *N*,*N*-dimethylformamide (DMF) and dimethyl sulfoxide (Table S1†). It instantly forms gels in a mixed solvent (DMF/H_2_O) with a suitable mixing ratio (Table S2†). CD spectral measurements indicated that most of the obtained gels were nearly CD-silent. Only a few assembly batches exhibited weak optical activities (Fig. S1a†). In addition, the SEM observations showed that both left- (*M*) and right-handed (*P*) helical structures were formed in the same sample (Fig. S1b†). These results indicated that gelation-induced symmetry breaking of BTACA occurred with a weak chiral bias. Magnetic stirring has been reported to be an effective approach for amplifying the chiral bias in symmetry-breaking systems.^33^,^39^–^43^ However, it is difficult to obtain homochiral assemblies by this method (see the mechanism discussion). Here, we investigate the application of a mechanical vortex mixing method by using a vortex mixer during the self-assembly process (see Fig. S2† for the experimental setup). Since gel formation was thermally reversible in DMF/H_2_O (1 : 1 v/v), the assembly and disassembly of BTACA can be easily manipulated by heating and cooling operations. If vortex mixing was continuously applied during the cooling process (also the self-assembly process) from 383 K to 298 K (about 10 minutes), a stable and well-dispersed suspension that exhibited strong and mirror-image CD signals can be obtained, as shown in Fig. 2a. Several spectral measurements, including X-ray diffraction, Fourier transform infrared spectroscopy, UV-Vis spectroscopy and fluorescence spectroscopy, were performed to investigate the self-assembled structures formed after this treatment (see Fig. S3 and S4† for more details). The sign and magnitude of the supramolecular chirality are quantified by the dissymmetry factor *g*_CD_, which is the ratio of CD intensity to the corresponding absorption (see the Methods section).^44^ The average *g*_CD_ was estimated to be ±0.043 at *λ* = 351 nm. However, if no external stimulation was applied during the cooling process, supramolecular gels were formed again with almost negligible CD signals, which was similar to the instant gels. Compared with the gel samples (Fig. S1a†), the amplification of the supramolecular chirality by vortex mixing is remarkable.

Since CD measurements might contain linear dichroism (LD) artifacts, particularly for achiral systems, the LD contribution was investigated (Fig. S5–S7†). According to the angle-dependent measurements, the contamination of the CD data by the LD artefact was evaluated to be 0.05% on the basis of a semi-empirical equation,^45^,^46^ indicating that the contribution of LD to the strong CD signals can be ignored.

Compared to the CD response, CPL is a unique property pertaining to the chiral luminous system, which can be used to evaluate the excited-state supramolecular chirality of an assembly. We further investigated the CPL of the BTACA assemblies, as shown in Fig. 2b. Although the monomers were achiral and CPL-silent, the assemblies formed through the vortex mixing showed strong CPL signals. The average absolute value of the dissymmetry factor *g*_lum_ for the assemblies was estimated to be 0.014, which is also a high value for organic systems.^47^

It should be emphasized that the vortex mixing-induced supramolecular chirality exhibited desirable repeatability and stability. As shown in Fig. 2c, the CD intensities of two independent samples were recorded after successive cycles of heating followed by vortex mixing treatment. Although the handedness was random, the strong and steady absolute *g*_CD_ values remained the same regardless of different handedness. More importantly, the CD intensity reached a steady maximum value after every vortex mixing treatment. As shown in Fig. 2d, the CD intensity remained almost constant across 40 different batches. Statistical analysis of the CD signals at 351 nm also showed that nearly half of the samples had a negative Cotton effect, while the other half displayed a positive Cotton effect after an identical vortex mixing treatment. So far, the symmetry breaking of achiral molecules in several self-assembly systems could be selected by purely physical fields, such as a hydrodynamic flow by stirring.^31^,^33^,^39^,^42^,^48^,^49^ However, the statistical distribution suggested that the chiral bias of the BTACA assemblies was not determined by the vortex mixing direction. In addition, the detection areas during CD measurement are also taken into consideration. Since a cuvette of 0.1 mm is used for measuring the CD spectra, a 30 μL suspension is enough for each CD measurement. As shown in Fig. S8,† we measured 20 times for one BTACA sample obtained by vortex mixing. Therefore, almost 600 μL suspension was measured for one sample. The effective detection area for CD measurement (JASCO J-1500 spectrometer) is about 0.5 cm^2^. Thus, about 10 cm^2^ area was measured for one sample. Clearly, there is no difference among these *g*_CD_ values. The as-prepared samples also displayed high stability even after storage for 17 days at room temperature (Fig. S9†).

**Fig. 3:**
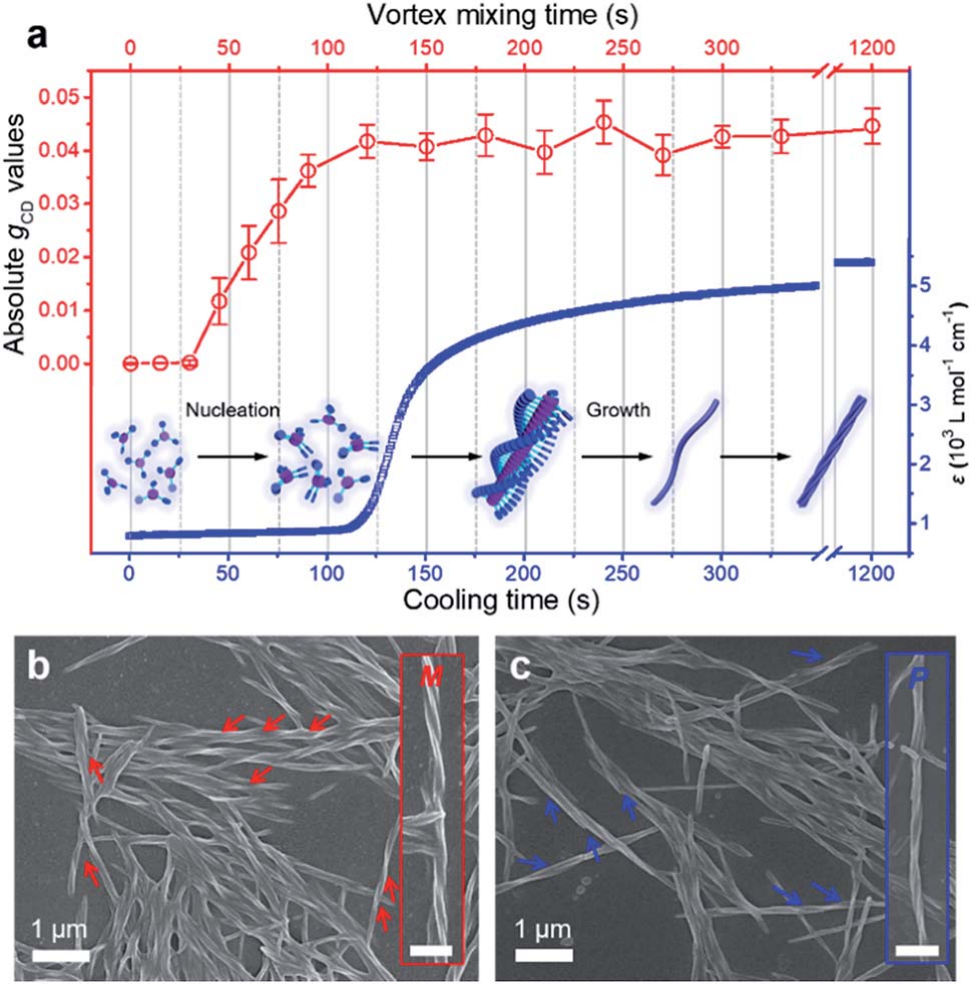
Time evolution and corresponding structures of the BTACA assemblies. (a) The absolute *g*_CD_ values of the BTACA assemblies prepared under varying vortex times (red curve) and the absorption data of aggregated molecules plotted as a function of cooling time (blue curve) in DMF/H_2_O (1 : 1 v/v) at 380 nm. Inset shows the possible route for this self-assembly. SEM images of the (b) M-type and (c) P-type helical structures after vortex mixing. The insets show the magnified helical structures. Scale bars, 500 nm.

For a better clarification of the vortex mixing-amplified supramolecular chirality in this achiral system, the influence of the vortex speed and time was investigated. By increasing the vortex speed, the absolute *g*_CD_ values of BTACA assemblies increased at first and subsequently levelled off (Fig. S10†). For the exploration of vortex mixing time, samples were firstly treated with various time periods at a fixed vortex speed of 2500 rpm, and then kept motionless for at least two hours before the CD measurement. As shown in Fig. 3a, the CD intensities increased rapidly and then reached a saturation point at 180 s. These results indicated that the supramolecular chirality reached the maximum values after approximately 3 minutes of stirring at 2500 rpm.

As a typical molecular model system, the assembly properties of BTAs have been extensively investigated in the past decade.^38^,^50^ One-dimensional (1D) helical stacks stabilized by three-fold intermolecular hydrogen-bonding and pi–pi stacking interactions are suggested for BTAs, which are also suitable for the assembly of BTACA. On the other hand, the mechanism of 1D supramolecular polymerization is classified into either cooperative/nucleation–elongation or isodesmic models.^51^ To study the emergence of supramolecular chirality in the system, we attempted to estimate their self-assembly by using the above reported models and their known features.^52^,^53^ It is known that the nucleation–elongation model involves two steps: nucleation and elongation in which elongation is a faster process than nucleation, while all binding constants throughout the course of polymerization are equal in an isodesmic mechanism. Therefore, an abrupt spectral change, caused by an abrupt change in monomer conversion, can be observed for the nucleation–elongation model, but this change is modest for the isodesmic model.^52^,^53^ As shown in Fig. 3a, we monitored the change in the absorption band at 380 nm, which corresponds to the aggregate absorption, during cooling of the hot solution (monomer state) to room temperature (assembled state). Clearly, the abrupt increase in the absorption of BTACA at approximately 120 s suggested that a non-isodesmic aggregation process occurred during the self-assembly process.^11^,^54^,^55^ Meanwhile, the temperature-dependent experiments also proved that the nucleation–elongation model could be applied to this system (Fig. S11†).^50^,^56^–^58^ Thus, one possible self-assembly route for BTACA molecules during the natural cooling process involved an initial nucleation stage within 120 s and a subsequent growth stage until room temperature was reached (Fig. 3a).

Since the vortex mixing was applied during the cooling, the vortex time and cooling time represent the same length of time. The comparative evaluation of both the CD and absorption intensities obtained at identical time clearly illustrated that vortex mixing can amplify the supramolecular chirality only during the process of nucleation. Through the real-time tracking of absorption spectra and *g*_CD_ values, we found that the vortex mixing during the nucleation stage is crucial.

To further confirm the enantiomeric excess, we carefully analysed the nanostructures of the BTACA assemblies. From the SEM images, only single-handed nanohelices can be observed in each sample. Specifically, the sample with a positive CD signal at 351 nm showed *M* helical structures (Fig. 3b), while the sample with a negative CD signal formed *P* helical structures (Fig. 3c). To confirm this result, more than one hundred SEM images were recorded within an area of 300 μm × 200 μm in the sample with a positive CD signal. All of the recognizable nanohelices in these images had *M* type handedness (Fig. S12†), suggesting an enantiomeric excess of 99.9% or above. These domains are much larger than those homochiral assemblies confirmed by scanning tunneling microscopy (STM).^59^,^60^ It should be noted that the determination of enantiomeric excess and the homochirality by using microscope observation has already been applied for chiral nanofibers and semiconductor helices.^59^–^62^ Here, combining the high enantiomeric excess evaluated by SEM and the stable yet repeatable CD intensities of the assemblies, we could conclude that a near-unity homochiral assembly was obtained from achiral BTACA molecules through a vortex mixing self-assembly.

### Converting racemic assemblies into a controllable homochiral state

**Fig. 4:**
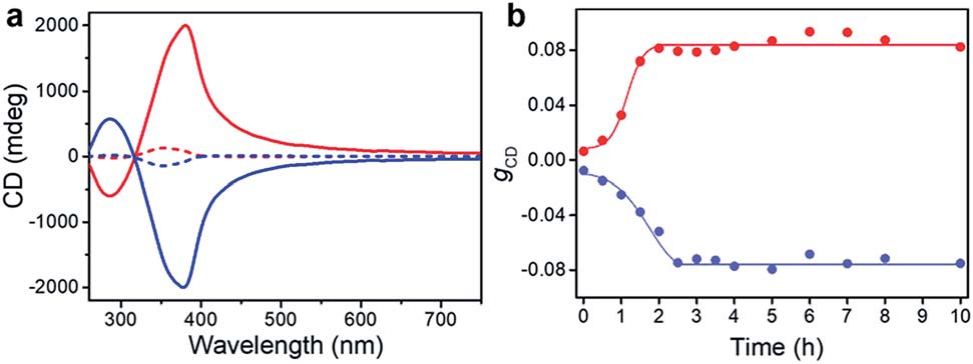
Ripening process of the racemic gels. (a) CD spectra of BTACA racemic gels with an initial 10% molar ratio of chiral seeds before (dash line) and after (solid line) the ripening process. (b) Evolution of *g*_CD_ for racemic samples with an initial 10% molar ratio of chiral seeds plotted against the ripening time at 333 K.

In the previous section we have shown that BTACA could self-assemble into homochiral assemblies using a vortex mixing treatment. However, the obtained supramolecular chirality was random and does not follow the vortex mixing direction. In addition, without the application of vortex mixing, only racemic gels were obtained. Therefore, we questioned whether we could obtain homochiral assemblies with determined chirality. We first tried this through the Viedma ripening method.^6^,^7^ Accordingly, the CD spectra of the racemic BTACA assemblies stirred with a magnetic bar and glass beads were recorded. However, unlike Viedma ripening, no CD activity was detected, even after 15 days of stirring (Fig. S13†). Alternatively, when a 10% molar ratio of the previously prepared homochiral assemblies was added into the racemic assemblies, the *g*_CD_ value was significantly enhanced from 0.0061 to 0.079 after a ripening process for 3.5 hours (Fig. 4a). It should be noted that the efficient ripening operation was carried out at 333 K, which is a temperature that does not destroy the assemblies (Fig. S14†). This temperature is also far below the complete disassembly temperature (383 K). Therefore, no nucleation occurred during the ripening process. From that perspective, the significantly increased chiral bias indicates that even the preformed racemic nanohelix can turn out to have the same chirality. More importantly, compared with the results from Fig. 2a and 4a, we obtained almost the same CD spectra, suggesting that both of them were reliable to obtain homochirality in supramolecular assemblies (Fig. S15†). Meanwhile, compared to vortex mixing treatment (Fig. 2a), the CD maximum is progressively red-shifted (from 351 nm to 380 nm), which might be caused by the entanglement of helical nanostructures after ripening (see Fig. S16 and S17† for more details). The higher *g*_CD_ values after ripening are because the absorption intensity is decreased along the longer wavelength, leading to a relatively higher ratio of CD to the absorption (see the definition of *g*_CD_ in the Methods section).

More important, the racemic gels with initial positive CD signals show amplified positive signals, while the racemic gels with initial negative CD signals show amplified negative signals. In other words, the final chirality of these complex systems is always consistent with that of the added assemblies. The initial homochiral assemblies appeared to work as chiral seeds, which can direct the chiral ripening of the racemic gels. The time dependence of the *g*_CD_ value shown in Fig. 4b revealed that the ripening-induced amplification of enantiomeric excess was a nonlinear process at the beginning (about 2.5 hours), subsequently reached a steady value, which is in good agreement with the complete chiral symmetry breaking in crystal growth.^6^,^63^ On the basis of these results, the determined homochirality of the racemic assemblies is suggested to be accomplished by following a ripening process in the presence of seeds from achiral molecules obtained *via* the vortex mixing self-assembly.

The effects of the temperature and initial enantiomeric excess in such a ripening process were also considered. At 293 K, the absolute *g*_CD_ value was still very low after an extended period of the ripening process with a 10% molar ratio of chiral seeds (Fig. S17 and S18†). This result might be due to the slow conversion of the different-handed assemblies at a low temperature. As shown in Fig. S19,† the *g*_CD_ values were recorded as a function of time for four individual experiments with various chiral seeds. Interestingly, only 1% molar ratio of chiral seeds can control the final chirality. However, low molar ratios of chiral seeds required relatively long ripening times (about 8 hours). These results are in good agreement with the previous reports in crystals,^6^,^63^ indicating that both the supramolecular assemblies and the crystallization might follow the same mechanism.

The main difference between the vortex mixing and ripening processes is the operating temperature. In the former case, vortex mixing is applied during the self-assembly from the monomers to supramolecular assemblies, where the temperature goes down from a higher temperature (in the molecular state) to room temperature (molecular assemblies). The significance of this process is the nucleation under vortex mixing (Fig. 3a). In contrast, the ripening procedure does not include the initial nucleation process, and it refers to the process that transfers the already formed racemic assemblies to a homochiral state, where the addition of the chiral seeds is important and necessary.

### Mechanism of vortex mixing-accompanied self-assembly

To reveal the mechanism of the induced homochirality, we provide a detailed discussion of each step of the vortex mixing-accompanied self-assembly (Fig. S20†). At the beginning, BTACA existed in a molecularly dispersed state in DMF solution, and thus, no CD signal was detected. After adding poor solvent (H_2_O), supramolecular gels with negligible CD activities were instantly formed. When the formed gels were heated to form a transparent solution and then subsequently cooled, supramolecular gels were formed again. However, neither the hot solution nor the resulting gels displayed recognizable optical activities. On the other hand, directly applying a vortex mixing treatment for the racemic gels at room temperature resulted in a well-dispersed but still CD-silent suspension. These experimental results indicate that without the application of vortex mixing, chirality was minimally produced. In addition, once the racemic assemblies were already formed, the vortex mixing was ineffective for enhancing the chirality.

**Fig. 5:**
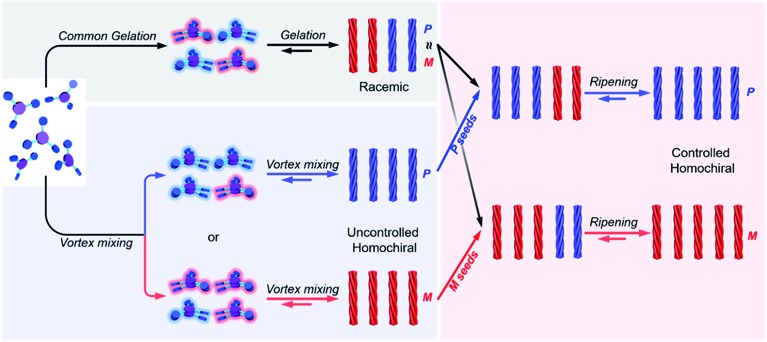
Schematic illustration of the mechanism towards homochirality. Common gelation only results in racemic gels due to the equal amount of two enantiomers whether in small nuclei or nanohelix stages. However, under continuous vortex, the achiral molecules self-assemble into small nuclei with nearly one handedness, which subsequently grow into larger homochiral nanohelices. Either *P* or *M* homochiral assemblies are generated because vortex cannot control the initial handedness of nuclei. On the other hand, the added homochiral seeds break the equilibrium between the *P* and *M* nanohelices in the racemic gels, and the homochiral state following the handedness of the seeds can be obtained finally *via* the ripening process.

As mentioned above, the formation of BTACA assemblies in DMF/H_2_O occurred in two stages: an initial nucleation process and a subsequent growth step (Fig. 3b). Primary nucleation can induce the conversion of achiral monomers into small chiral aggregates *via* non-covalent interactions. From the perspective of energy, the two enantiomers, *M* and *P* dimers, were indistinguishable, and thus, they existed with equal probabilities.^63^ These dimers gradually grew by capturing neighbouring achiral molecules to form chiral assemblies. Since the primary nucleation of the *M* and *P* assemblies was equivalent, a racemic gel was obtained finally. This is the common gelation process, as shown in Fig. 5. When vortex mixing treatment was applied during self-assembly, the rapid generation of secondary nuclei reduced the concentration of monomers to a level at which the primary nucleation is suppressed. In addition, the primary nucleation is a relatively cumbersome process due to the kinetic barrier of reaching the critical nucleus. Therefore, the formation of nuclei of the opposite handedness is possible due to the competition caused by vortex mixing. As a result, one enantiomorph occasionally dominated the system, and the population of the chiral bias was gradually enhanced. However, there was no preference for the chiral bias. After that, the subsequent supramolecular assembly can further amplify the initial chirality to give a homochiral polymer with either an *M* or a *P* handedness. The initial single chirality of the nuclei that was induced by vortex mixing was vital in this case.

The remarkable difference between our work and Viedma ripening is that direct vortex mixing or stirring cannot amplify the chiral bias of racemic gels due to the dynamic properties of supramolecular assemblies and the weak chiral bias caused by symmetry breaking. However, the addition of enantiopure seeds can break the equilibrium between the *P* and *M* assemblies, and homochiral systems with desired handedness finally obtained *via* a ripening process. During this deracemization process, a mechanism similar to attrition-enhanced Ostwald ripening should not be neglected, which might remove the competing lineages of two enantiomers, leaving the seed-determined ancestor for the entire system.^64^,^65^

## Conclusions

Achieving single-handedness with achiral molecules is vital in nature. Here, we proposed an effective strategy based on vortex mixing-accompanied self-assembly for achieving near-unity homochirality from exclusively achiral molecules. By applying vortex mixing during the self-assembly process, the spontaneous symmetry-breaking could be amplified, thus achieving single-handed supramolecular assemblies. Different from previously reported examples, the direction of vortex mixing could not select the chirality in the present case. When chiral seeds were present, homochirality can be obtained in a deterministic manner *via* ripening operation. Thus, combining these two processes, we ultimately obtained not only controlled but also near-unity homochiral entities by self-assembly of achiral molecules. The results given here support the deterministic evolution of supramolecular handedness.

## Conflicts of interest

There are no conflicts to declare.

## Supplementary Material

Supplementary information
