# Supplementary material for: Towards homochiral supramolecular entities from achiral molecules by vortex mixing-accompanied self-assembly
Source: Chem Sci. 2019 Jan 7;10(9):2718–24. doi: 10.1039/c8sc04687e (PMC6419933; doi:10.1039/c8sc04687e)
Supplement: Supplementary file 1 [file SC-010-C8SC04687E-s001.pdf]

Electronic Supplementary Material (ESI) for Chemical Science.
This journal is © The Royal Society of Chemistry 2019

Electronic Supplementary Information (ESI)

Towards homochiral supramolecular entities from achiral molecules by vortex mixing-accompanied self-assembly

Yutao Sang, Dong Yang, Pengfei Duan* and Minghua Liu*

*Correspondence to: e-mail: duanpf@nanoctr.cn (P. D.); liumh@iccas.ac.cn (M. L.).

The PDF file includes:

1. Experimental section

2. Synthesis and characterization of compounds

3. Supplementary Tables and Figures

Table S1-S2.

Figure S1-S20

4. Supplementary SEM images

1. Experimental section

Materials

All the starting materials and solvents were obtained from commercial suppliers and used as received. Milli-Q water (18.2 M·cm) was used in all cases. 1,3,5-Benzenetricarbonyl trichloride was purchased from Acros. Ethyl 4-aminocinnamate was purchased from Alfa Aesa. It is essential to use new glassware, beads, and stirring bars for each experiment to avoid chiral contamination.

General methods

^{1}H NMR spectra were recorded on a Bruker AV400 (400 MHz) spectrometer. Mass spectral data were obtained by using a BIFLEIII matrix-assisted laser desorption/ionization time of fight mass spectrometry (MALDI-TOF MS) instrument. Elemental analysis was performed on a Carlo–Erba-1106 Thermo-Quest. Scanning electron microscopy (SEM) was performed on a Hitachi S-4800 FE-SEM with an accelerating voltage of 10 kV. Before SEM measurement, the samples on silicon wafers were coated with a thin layer of Pt to increase the contrast. FT-IR spectra were recorded on a JASCO FT/IR-660 plus spectrophotometer with the resolution of 4 cm^{-1} at room temperature. Samples were vacuum-dried and made into plates with KBr for FT-IR spectral measurements. X-ray diffraction (XRD) analysis was performed on a Rigaku D/Max-2500 X-ray diffractometer (Japan) with Cu Kα radiation (λ=1.5406 Å), which was operated at a voltage of 40 kV and a current of 200 mA. Fluorescence and UV-Vis spectra were recorded on Hitachi F-4500 fluorescence spectrophotometer and JASCO UV-550 spectrometer, respectively.

Chiroptical analysis

Circular dichroism (CD) and linear dichroism (LD) spectra were obtained by using JASCO J-1500 spectrometers. Circularly polarized luminescence (CPL) measurements were performed with a JASCO CPL-200 spectrometer. The magnitude of the circular polarization at the ground state is defined as $g_{CD} = 2 \times (\varepsilon_L - \varepsilon_R)/(\varepsilon_L + \varepsilon_R)$, where ε_L and ε_R refer to the extinction coefficients for left- and right-

handed circularly polarized light, respectively. Experimentally, the value of g_{CD} is defined as g_{CD} = [ellipticity/32980]/absorbance at the CD extremum. The magnitude of CPL can be evaluated by the luminescence dissymmetry factor (g_{lum}), which is defined as $g_{lum} = 2 \times (I_L - I_R)/(I_L + I_R)$, where I_L and I_R refer to the intensity of left- and right-handed CPL, respectively. Experimentally, the value of g_{lum} is defined as g_{lum} = [elipticity/(32980/ln10)]/total fluorescence intensity at the CPL extremum. For the measurement of CD spectra, the cuvette was placed perpendicularly to the light path of CD spectrometer and rotated within the cuvette plane in or-der to rule out the possibility of the birefringency phenomena and eliminate the possible angle dependence of the CD signals. To estimate the contribution of LD effect on the true CD signal, 36 CD and LD spectra of the samples were measured in steps of 10° by rotating the sample which fixed in the homemade rotator.

Vortex mixing treatment

5 mg BTACA was first dissolved in 0.7 mL dimethylforma-mide (DMF) by sonication in a 5 mL vial, then 0.7 mL Mil-li-Q water was injected with a pipette and instant gels were formed in the mixed solvent. Upon heating (383 K), the gels became into transparent solution. Then vortex mixing at 2500 rpm was applied to the hot solution for 10 minutes. After that, white suspensions with good dispersion were obtained.

Ripening treatment

In a typical procedure, a 5.0 ×10.0 mm Teflon-coated magnetic bar, 620 mg glass beads (ø =1.5-2.0 mm) and different amount of chiral seeds (1%, 2.5%, 5% and 10% molar ratio) that prepared by vortex mixing process were added into the racemic gels. After that, these samples were placed in oil bathes at different temperature and stirred at 1000 rpm.

2. Synthesis and characterization of compounds

The BTA core with three functional cinnamic acid side chains (BTACA) was prepared by the hydrolysis of the ethyl cinnamate-substituted 1,3,5-benzenetricarboxamide (BTACE).

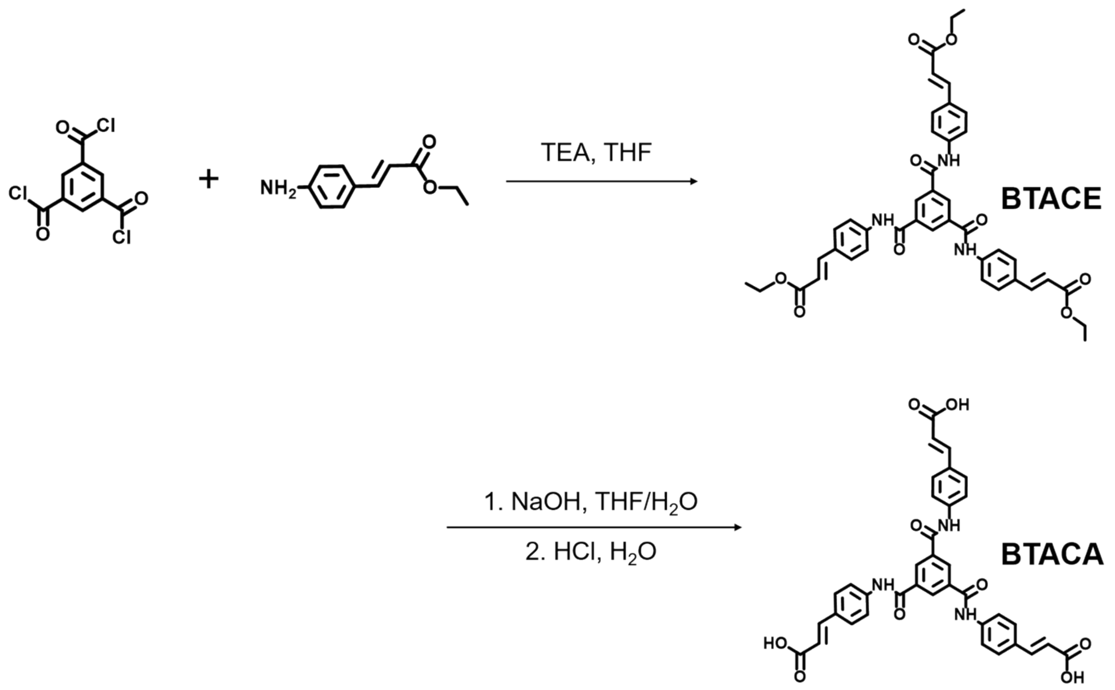

Briefly, BTACE was firstly prepared by treating 1, 3, 5-benzenetricarbonyl trichloride with ethyl 4-aminobenzoate. 1,3,5-benzenetricarbonyl trichloride (1.06 g, 4.0 mmol) was treated slowly with excess ethyl 4-aminocinnamate (3.06 g, 16.0 mmol) and trimethylamine (4.4 mL, 32.0 mmol) in dry THF at 0 ℃ and stirred overnight at ambient conditions. The reaction mixture was filtered and then concentrated by rotary evaporation. After adding 100 mL methanol to the residue, the solid precipitate was filtered and washed consecutively with water and methanol. The resultant powder was dried to give a white powder with a yield of 88%.

2 mL 4 M NaOH aqueous solution was added slowly to 40 mL BTACE (1.46 g, 2 mmol) suspension in THF/H_2O (1/1, v/v) and stirred overnight at room temperature. The obtained yellow solution was added 60 mL water and treated with 1 M dilute HCl until pH=1 and solid precipitate was obtained. After filtration, the solid was washed

with water and methanol to get the final product with a yield of 73%. ^{1}H NMR (400MHz, d_6-DMSO, 298K, ppm): 6.46-6.50 (d, 3H, CH), 6.56-6.60 (d, 3H, CH), 7.73-7.75 (d, 6H, Ar-H), 7.89-7.91(d, 6H, Ar-H), 8.73 (s, 3H, Ar-H), 10.78 (s, 3H, N-H), 12.31 (s, 3H, O-H). MALDI-TOF MS: calcd. for $C_{36}H_{27}N_3O_9$, M^+: m/z=645.17; found M^+: m/z=645. Elemental Analysis: calcd. for $C_{36}H_{27}N_3O_9 \cdot H_2O$: C 65.16, H 4.40, N 6.33; found: C 65.35, H 4.65, N 6.28.

3. Supplementary Tables and Figures

Table S1. Solubility of BTACA in various solvents

Solvent	Phase [a]
H_2O	I
ethyl acetate	I
methanol	I
ethanol	I
THF	I
DMF	S
DMSO	S
acetonitrile	I
$CHCl_3$	I
CH_2Cl_2	I
acetone	I
toluene	I
n-hexane	I
cyclohexane	I

[a] S: soluble; I: insoluble

Table S2. The effect of DMF/H_2O volume ratio on the gelation properties of BTACA.

Volume ratio [a]	Phase [b]
10-0	S
9-1	S
8-2	S
7-3	PG
6-4	G
5-5	G
4-6	PG
3-7	PG
2-8	PG
1-9	PG
0-10	I

[a] The total volume of DMF/H_2O is 1.4 mL and the concentration of BTACA is 5.54 mM.

[b] S: solution; PG: partial gel; G: stable gel; I: insoluble.

a
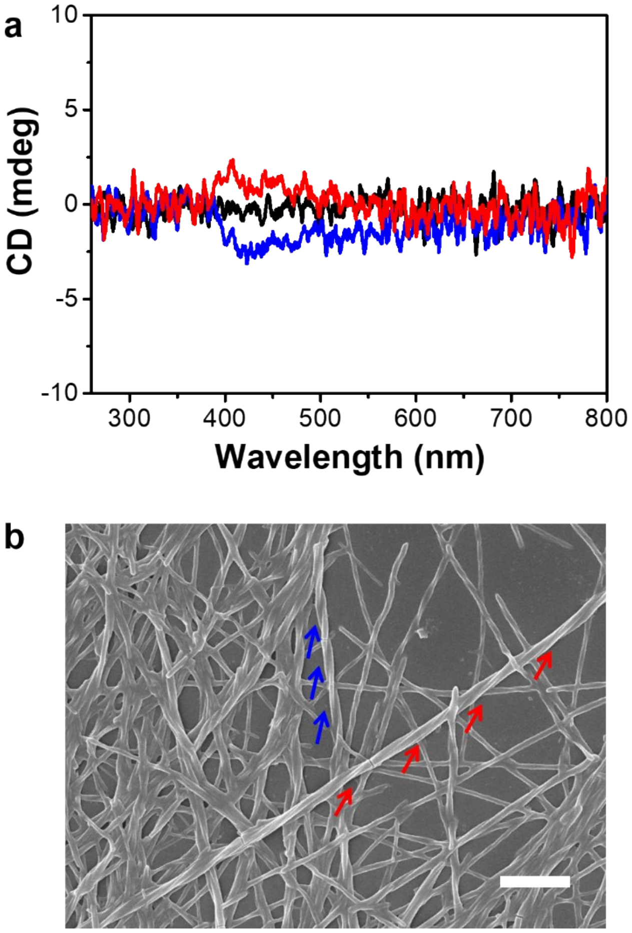

Fig. S1. Characterization of supramolecular gels. CD spectra (**a**) and SEM image (**b**) of BTACA gels (5.54 mM) in DMF/H_2O (1:1 v/v). Scale bars, 1 μm.

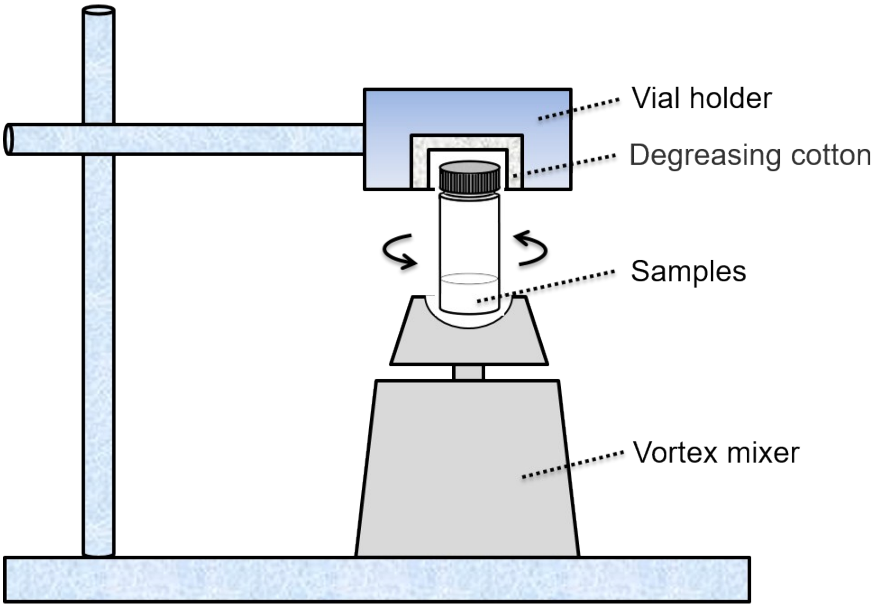

Fig. S2. Schematic showing the experimental setup for vortex mixing treatment by using vortex mixer. The vortex mixing treatment was achieved by home-made devices, which contains a vortex mixer (MX-S, DLAB Scientific Co., Ltd.) with adjustable speed, cantilever support and sample holder.

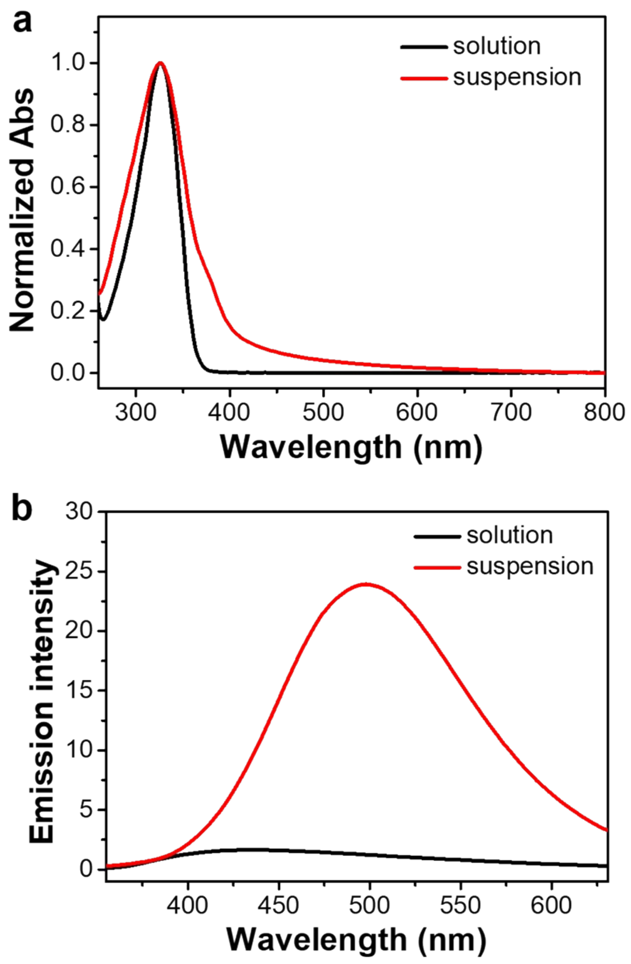

Fig. S3. Absorption and fluorescence spectra of BTACA. Absorption (**a**) and fluorescence spectra (**b**) of BTACA DMF solution and suspension in DMF/H_2O (1:1 v/v). BTACA concentration: 5.54 mM.

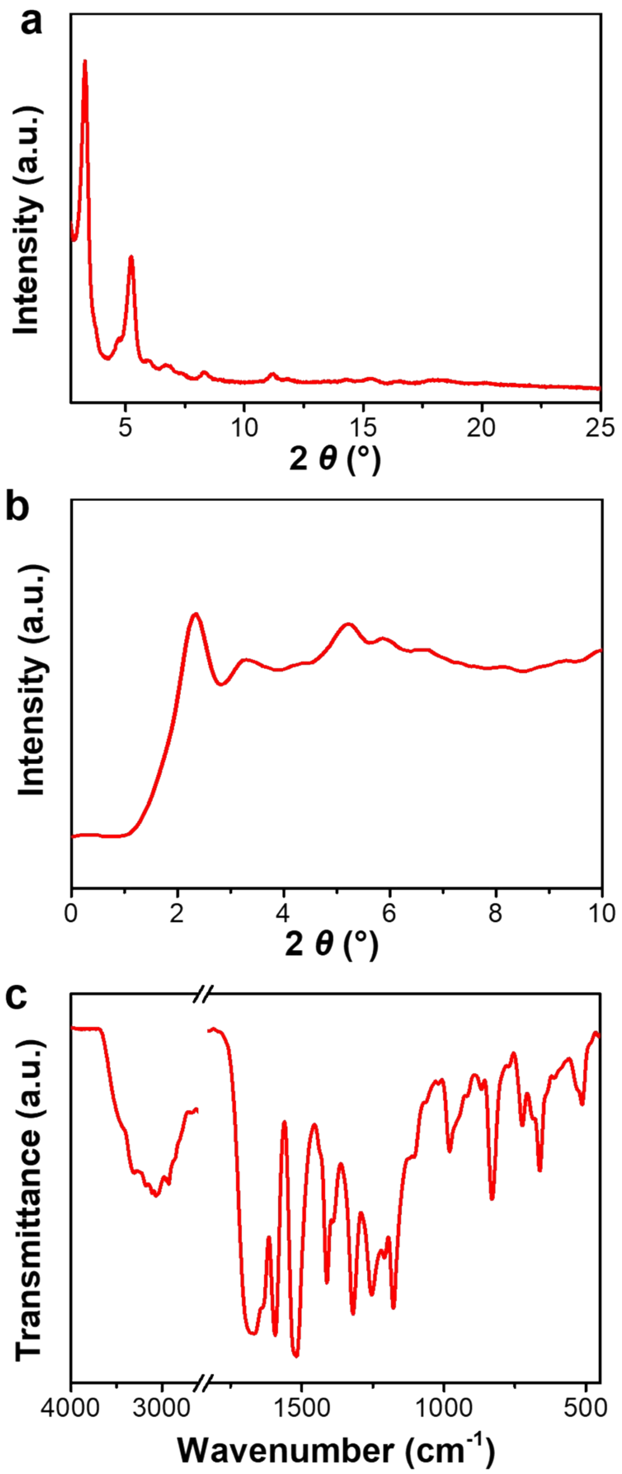

Fig. S4. Characterization of BTACA assemblies after vortex mixing process. (**a**) Power XRD of xerogel of BTACA. (**b**) Grazing incidence wide-angle X-ray scattering (GIWAXS) profiles of BTACA assemblies in DMF/H_2O (1:1 v/v). (**c**) FTIR spectra of xerogel of BTACA.

The assemblies of BTACA in DMF/H_2O through vortex mixing were characterized by means of X-ray diffraction (XRD), UV-Vis and fluorescence spectral measurements. Compared with its solution, UV-Vis spectra of BTACA assemblies broadened with a shoulder peak at 380 nm which suggested the existence of *J*-like aggregation (**Fig. S3a**). Meanwhile, the fluorescence intensity was significantly enhanced, accompanying with a drastic red-shift from 434 to 498 nm after assembly (**Fig. S3b**). The XRD profile of BTACA xerogel showed a series of peaks at 2.29°, 4.68°, 5.21° and 6.82°, and corresponding distances were estimated to be 3.85, 2.70, 1.88 and 1.69 nm, respectively (**Fig. S4a**). This indicates that the molecular packing adopts a body-centred cubic structure pattern. Moreover, the in situ investigation of BTACA assemblies in DMF/H_2O (1:1 v/v) showed identical peaks, which indicated the highly ordered molecular packing (**Fig. S4b**). Driving force of supramolecular assembly could be further confirmed by Fourier transform-infrared (FTIR) measurement. The peak at 1666 cm^{-1} for BTACA clearly indicated the formation of hydrogen bond from carboxylic acid groups (**Fig. S4c**) while the peaks at 1593 cm^{-1} (amide I band) and 1518 cm^{-1} (amid II band) suggested the existence of C=O and N–H in the hydrogen bond form. FTIR data demonstrated that the hydrogen bond between carboxylic acid groups played a crucial role in the supramolecular assembly of BTACA.

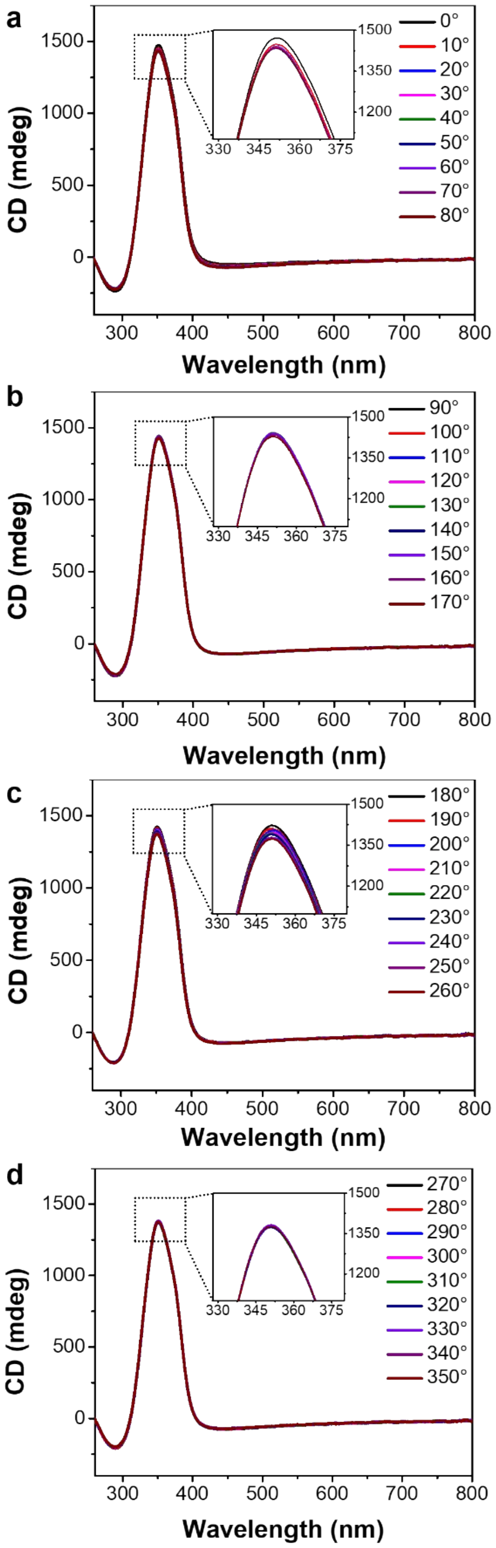

Fig. S5. Angle-dependent CD measurements. 36 CD spectra of BTACA (5.54 mM) suspension in DMF/H_2O (1:1 v/v) when rotating the sample about the optical axis in steps of 10°: 0°~ 80° (**a**), 90°~ 170° (**b**), 180°~ 260° (**c**), 270°~ 350° (**d**). When the rotation angle varied from 0° to 350° with a step of 10°, the 36 individual CD spectra showed different CD amplitude. This result suggests that there exists little LD effect in the observed CD spectra.

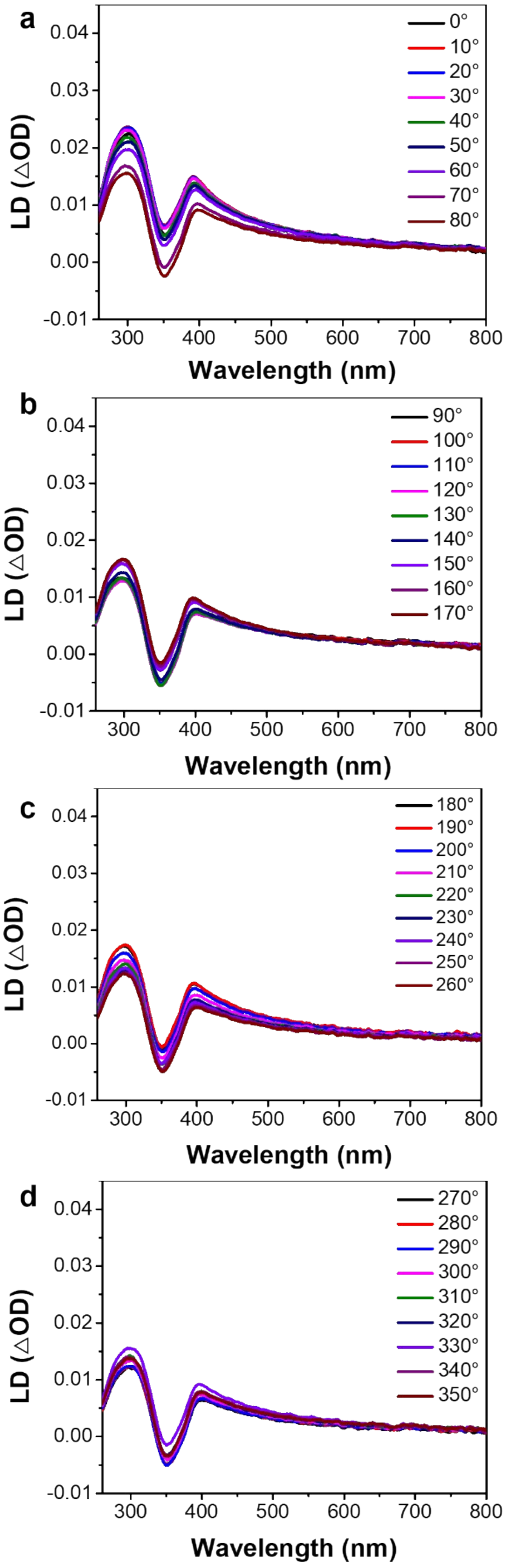

Fig. S6. Angle-dependent LD measurements. 36 LD spectra of of BTACA (5.54 mM) suspension in DMF/H_2O (1:1 v/v) when rotating the sample about the optical axis in steps of 10°: 0°~ 80° (**a**), 90°~ 170° (**b**), 180°~ 260° (**c**), 270°~ 350° (**d**). The test condition was the same as that for CD measurement in **Fig. S5**. The obtained 36 LD spectra showed different LD curves, which indicated that there exists macroscopic anisotropic in the suspension and thus results in LD artifacts.

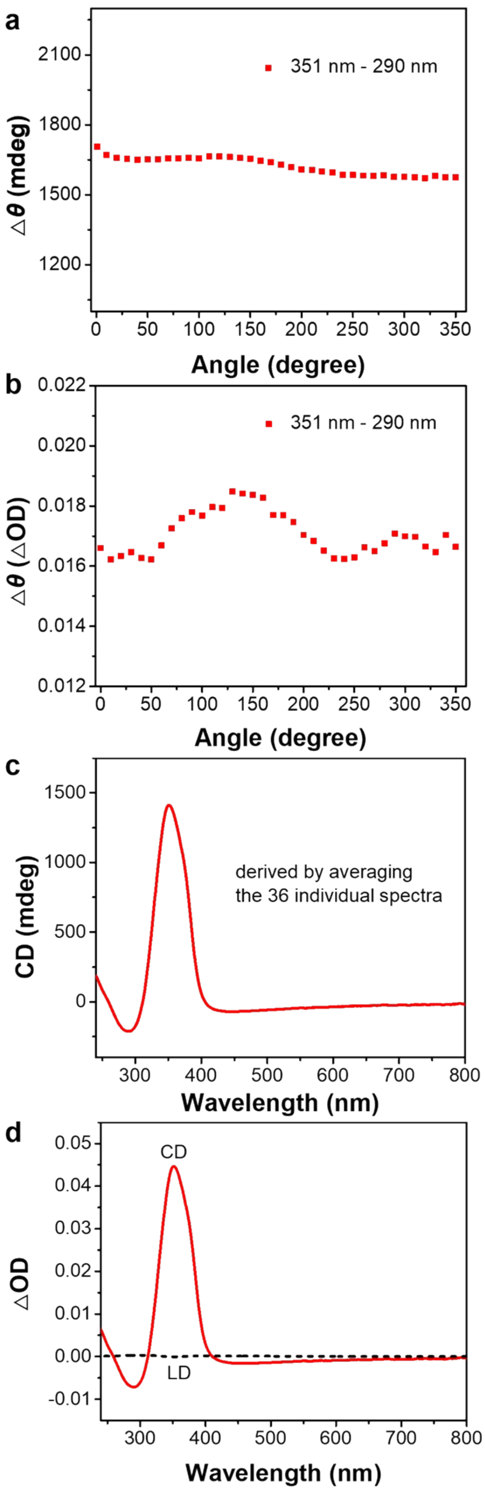

Fig. S7. Analysis of the angle-dependent CD and LD spectra. (**a**) Angle-dependent CD difference from the maximum value (351 nm) to minimum value (290 nm) extracted from the 36 CD spectra in **Fig. S5**. (**b**) Angle-dependent LD difference from the maximum value (351 nm) to the minimum value (290 nm) extracted from the 36 LD spectra in **Fig. S6**. (**c**) The mean spectrum of all 36 CD spectra. (**d**) The mean spectrum of all 36 CD spectra with the unit ΔOD for comparison (solid line) with the mean spectrum of all 36 LD spectra (dash line).

In **Fig. S7a**, the CD difference from the maximum value (351 nm) to the minimum value (290 nm) fluctuated around 1600 mdeg. This nonzero value suggested that the observed CD signal represents the authenticity of helical chirality in the suspension system. On the contrary, the values of 36 individual LD spectra fluctuated with the testing angle, suggesting that the angle-dependent LD effect can be eliminated by averaging all of the LD spectra (**Fig. S7b**). Therefore, the true CD intensity can be obtained by averaging all of these 36 individual spectra as shown in **Fig. S7c**. Moreover, the contribution of LD to the CD spectra could be quantitatively estimated by using the following semi-empirical equation (*45*):

$$\text{Contamination of CD by LD} = LD \times 0.02 / \text{CD observed}$$

The contamination of CD by LD in the present case was estimated about 0.05%.

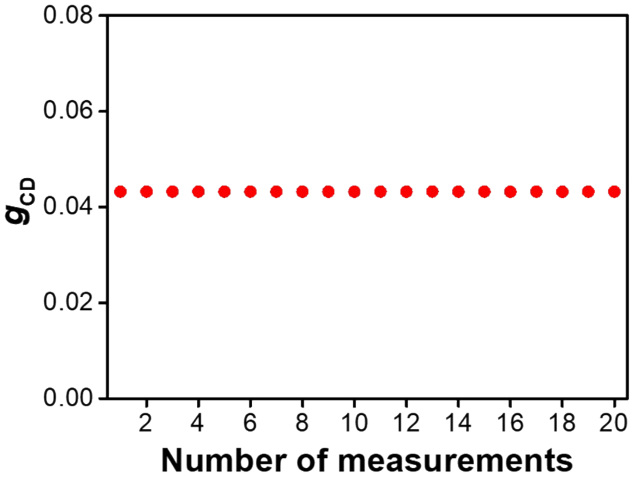

Fig. S8. Statistical distribution of g_{CD} values from one BTACA samples obtained by vortex mixing process. BTACA concentration: 5.54 mM in DMF/H_2O (1:1 v/v).

The detection areas during CD measurement are also taken into consideration. Since cuvette of 0.1 mm is used for measuring the CD spectra, 30 μL suspension is enough for each CD measurement. As shown in **Fig. S8**, we measured 20 times for one BTACA samples obtained by vortex mixing. Therefore, almost 600 μL suspension were measured for one sample. On the other hand, the effective detection areas for CD measurement (JASCO J-1500 spectrometer) is about 0.5 cm^2 (the diameter is 8 mm). Thus, about 10 cm^2 area was measured for one sample. Clearly, there is no difference among them, indicating the well-dispersed and homochiral of BTACA assembles after vortex mixing process.

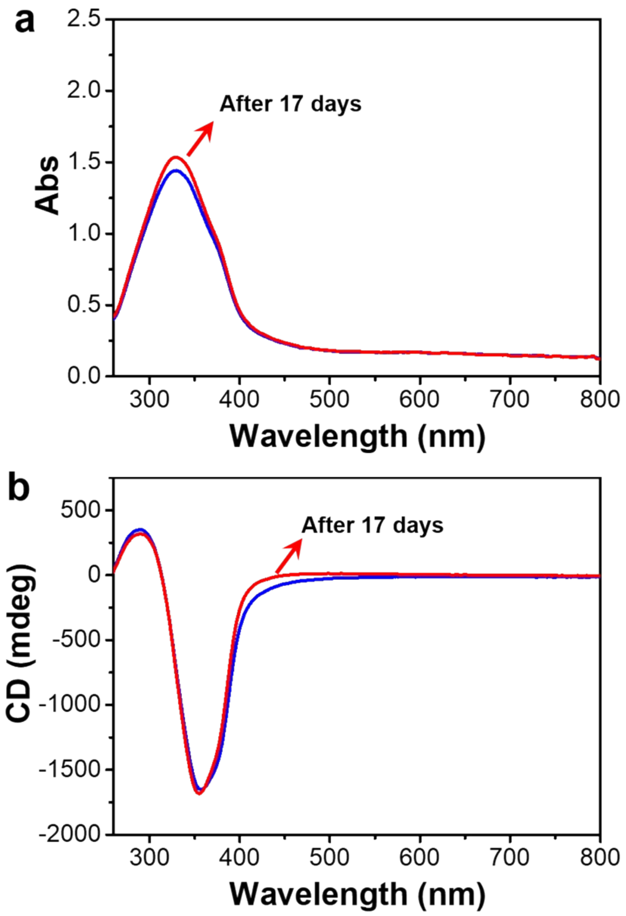

Fig. S9. Stability of BTACA assemblies after vortex mixing process at room temperature. UV-vis (**a**) and CD (**b**) spectra of BTACA assemblies before (blue line) and after (red line) 17 days. BTACA concentration: 5.54 mM in DMF/H_2O (1:1 v/v).

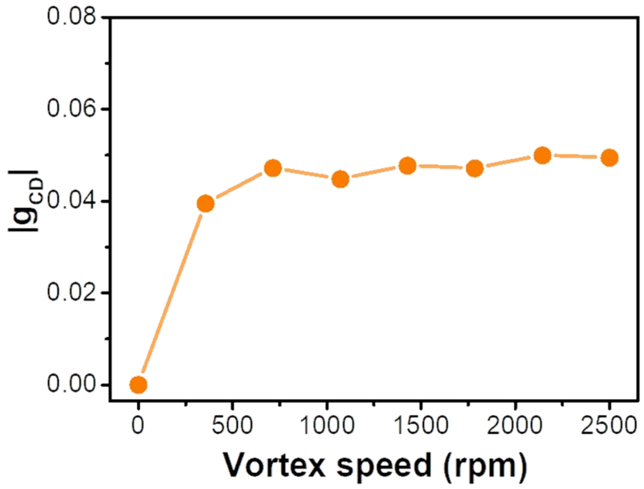

Fig. S10. The |g_{CD}| values of BTACA assemblies under diverse vortex mixing speeds.

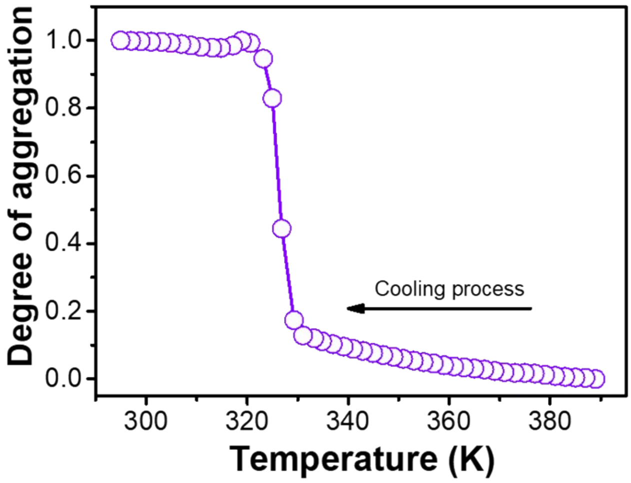

Fig. S11. Plot of degree of aggregation against temperature (283-388 K) for BTACA (5.54 mM) in DMF/H_2O (1:1 v/v).

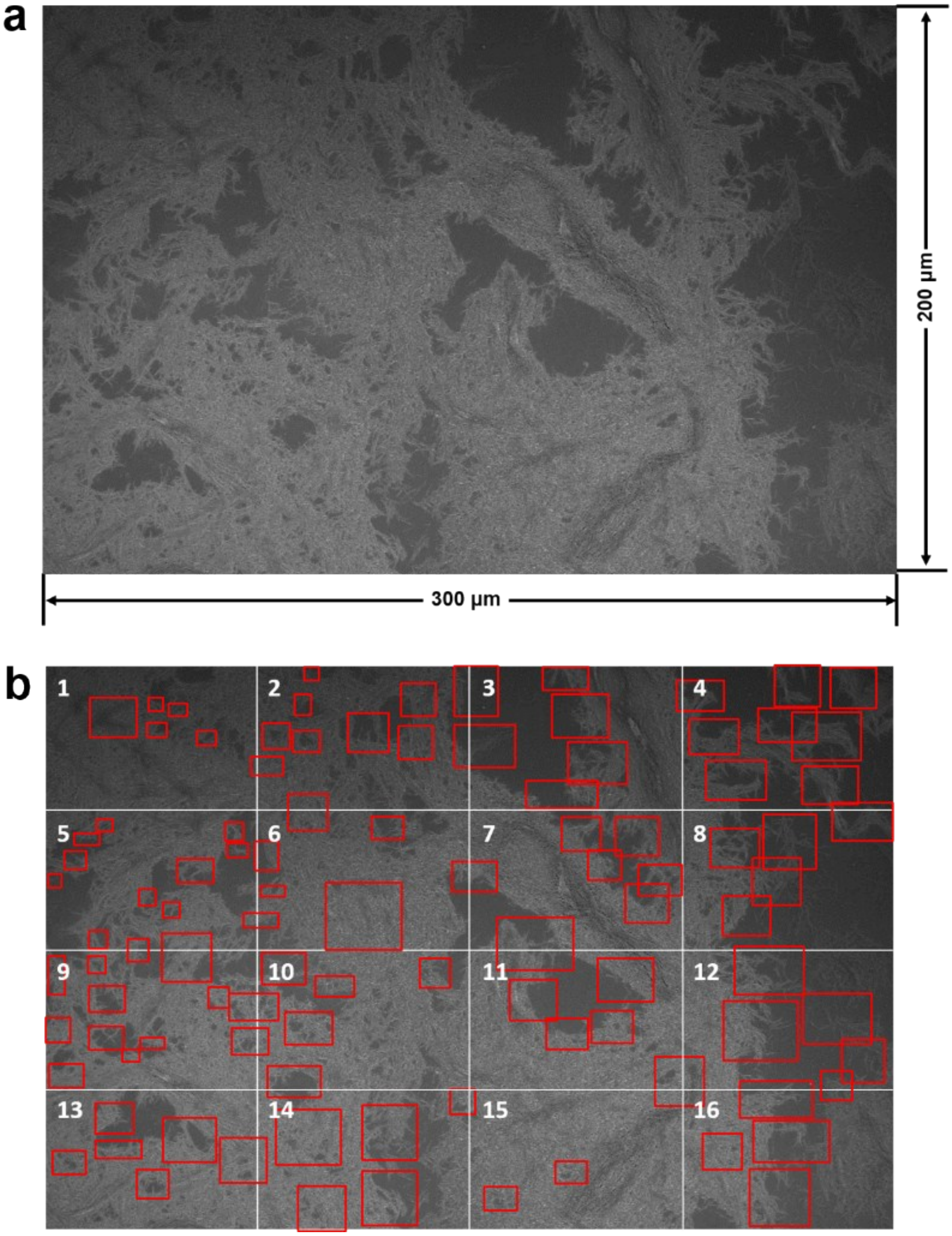

Fig. S12. SEM images of BTACA suspensions after vortex mixing process. SEM images in (**a**) shows the same SEM images as in (**b**) but without the markers. Only *P* handed nanohelix were observed within the scope of 300 µm × 200 µm. The red boxes in (**b**) represented that the scopes were carefully analyzed, which were listed in the end of this file.

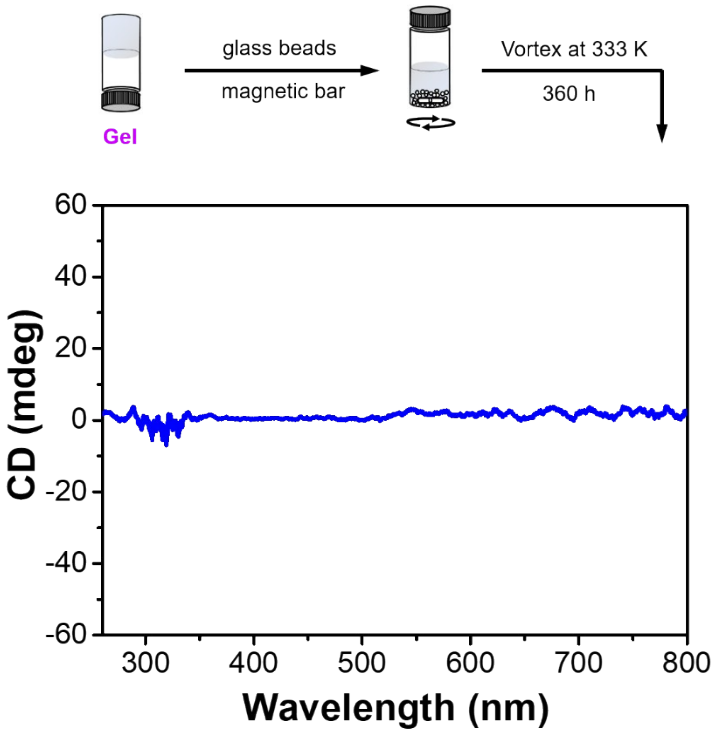

Fig. S13. CD spectra of BTACA racemic gels after vortex stirring for 360 h at 333 K and 1000 rpm. BTACA concentration: 5.54 mM in DMF/H_2O (1:1 v/v). The vortex stirring was achieved by using magnetic bar and beads.

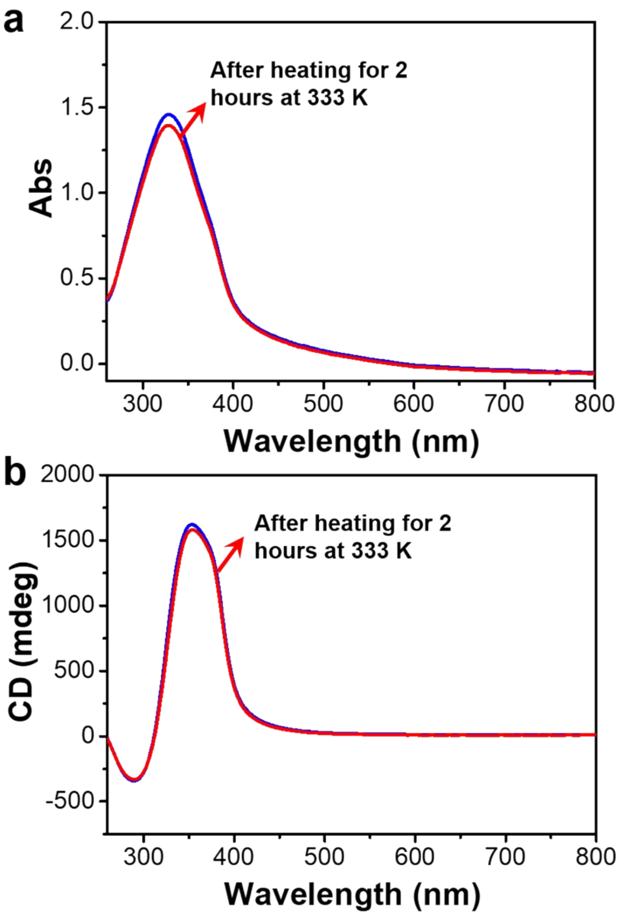

Fig. S14. Stability of BTACA assemblies at 333 K. CD spectra of BTACA assemblies before (bule line) and after (red line) heating for 2 hours at 333 K. BTACA concentration: 5.54 mM in DMF/H_2O (1:1 v/v).

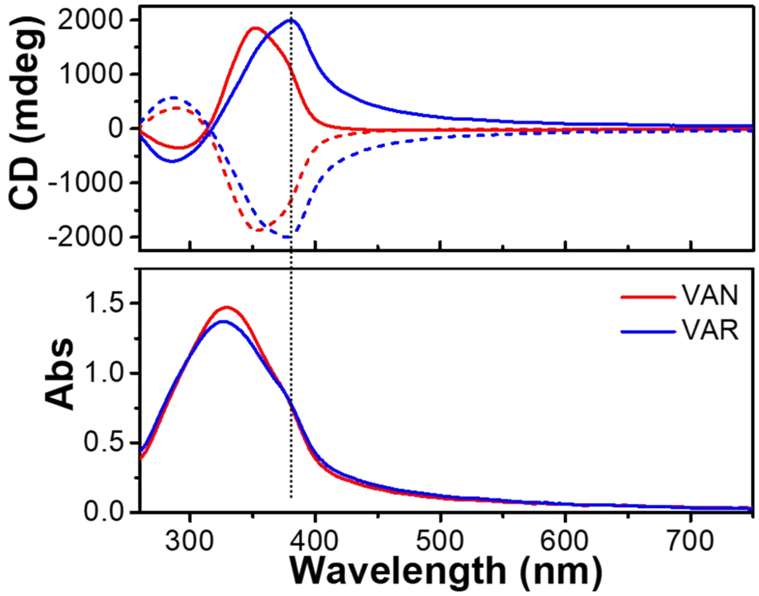

Fig. S15. The comparison of BTACA assemblies after vortex mixing (red line) and ripening (blue line) processes. (**Top**) CD spectra, (**Bottom**) corresponding UV-vis absorbance spectra.

By carefully looking the UV-vis absorbance spectra of the BTACA assemblies after vortex mixing (**Fig. S3a**), we could see a small shoulder peak at 380 nm besides the main peak at 328 nm. This shoulder peak can be attributed to the *J*-like aggregation (pi-pi stacking) of BTACA assembles. After the vortex mixing process, the assemblies were slender. With further ripening operation, the assemblies grew larger, which was reflected by a slight decrease of the main absorption peak and the entanglement of helical nanostrucutres (**Fig. S15**).

In the CD spectra, due to the formation of the chiral nanostructures, both of these species contributed to the CD signals. For the vortex mixing sample, the CD peak showed at 351 nm, while for the ripening processed assemblies, the CD maximum moved to 380 nm, which was the right position of shoulder absorption peak. In any cases, we saw that the crossover is in the same place at 313 nm (the position of the chromophore), indicating that the exciton coupling is based on the interactions between these chromophores after aggregation.

Considering that the ripening process needed much longer ripening time, these change in CD spectra might be caused by the enhanced *J*-like aggregation after thermodynamic equilibrium. In addition, tiny nanostructure difference of BTACA assemblies between these two process were observed from the SEM images (**Fig. 3 and S16**), which might also lead to the different CD spectra.

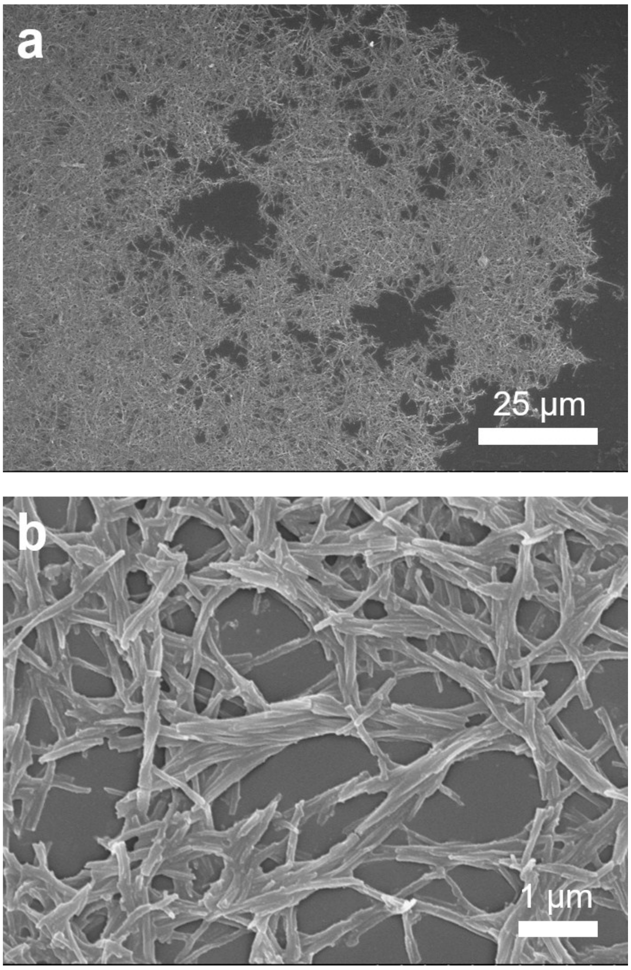

Fig. S16. SEM images of the BTACA assemblies after ripening process for 6 hours.

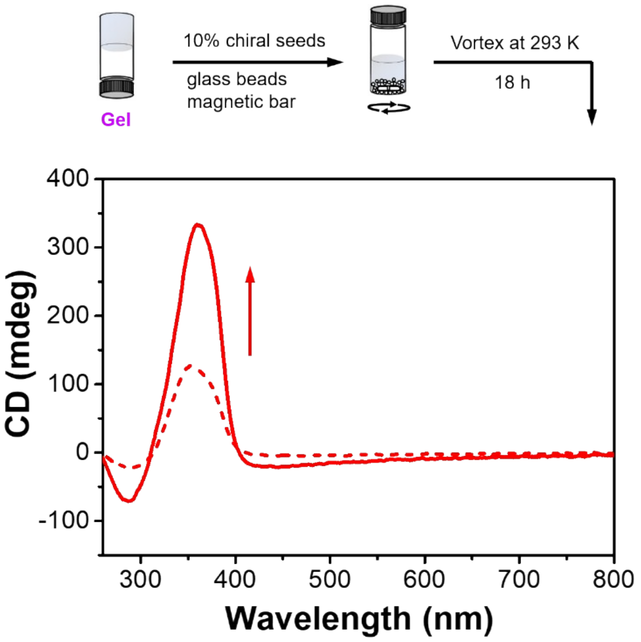

Fig. S17. CD spectra of BTACA racemic gels with 10% molar ratio of chiral seeds before (dash line) and after (solid line) vortex stirring for 18 h at 293 K and 1000 rpm. BTACA concentration: 5.54 mM in DMF/H_2O (1:1 v/v). The vortex stirring was achieved by using magnetic bar and beads.

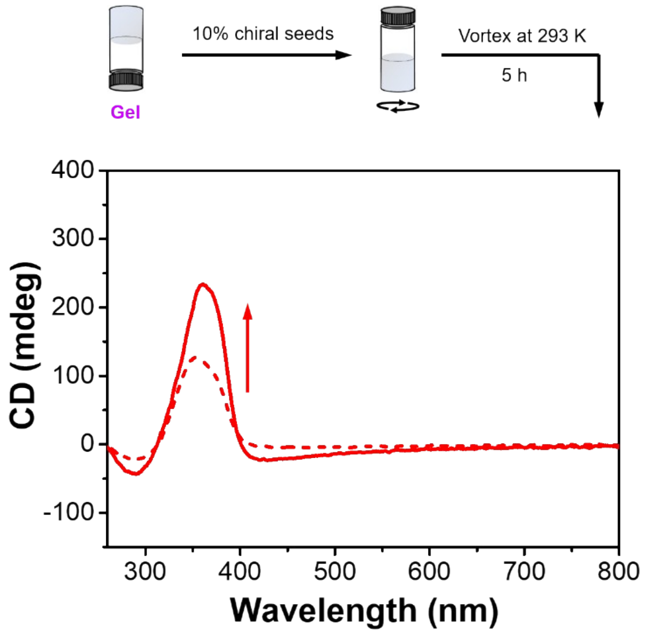

Fig. S18. CD spectra of BTACA racemic gels with 10% molar ratio of chiral seeds before (dash line) and after (solid line) vortex mixing for 5 h at 293 K and 2500 rpm. BTACA concentration: 5.54 mM in DMF/H_2O (1:1 v/v). The vortex mixing was achieved by using vortex mixer.

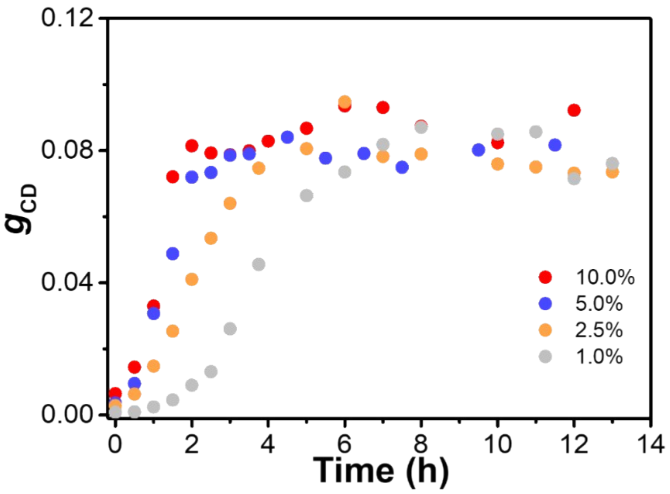

Fig. S19. Evolution of g_{CD} for BTACA gels with various initial molar ratio of chiral bias against vortex stirring time at 333 K. The vortex stirring was achieved by using magnetic bar and beads.

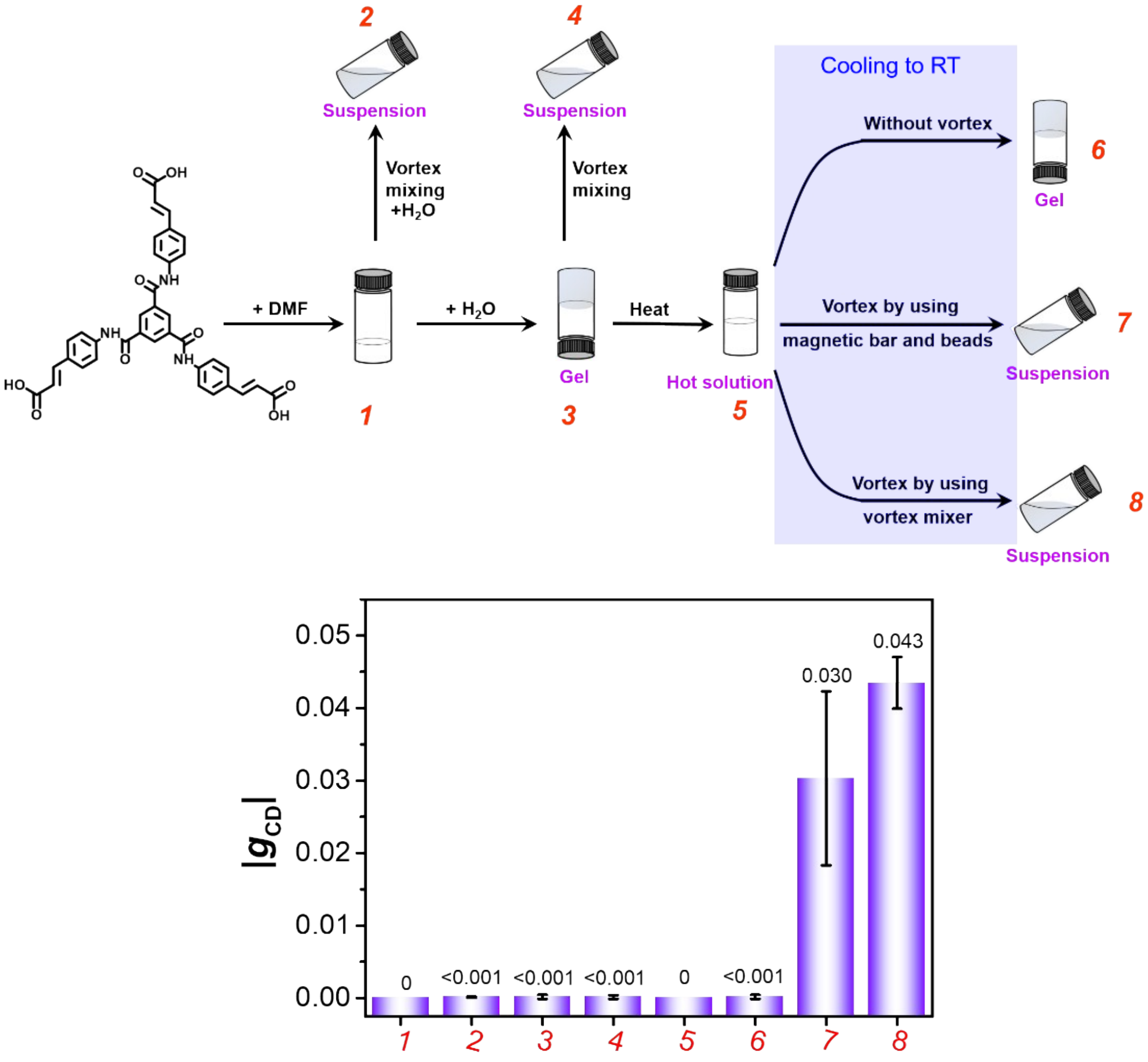

Fig. S20. Evaluation of each step during vortex procedure. Schematic representation of vortex procedure by using vortex mixer and magnetic bar and beads (**top**). Corresponding $|g_{CD}|$ values of BTACA samples after different treatment (**bottom**). BTACA concentration: 5.54 mM in DMF/H_2O (1:1 v/v).

4. Supplementary SEM images

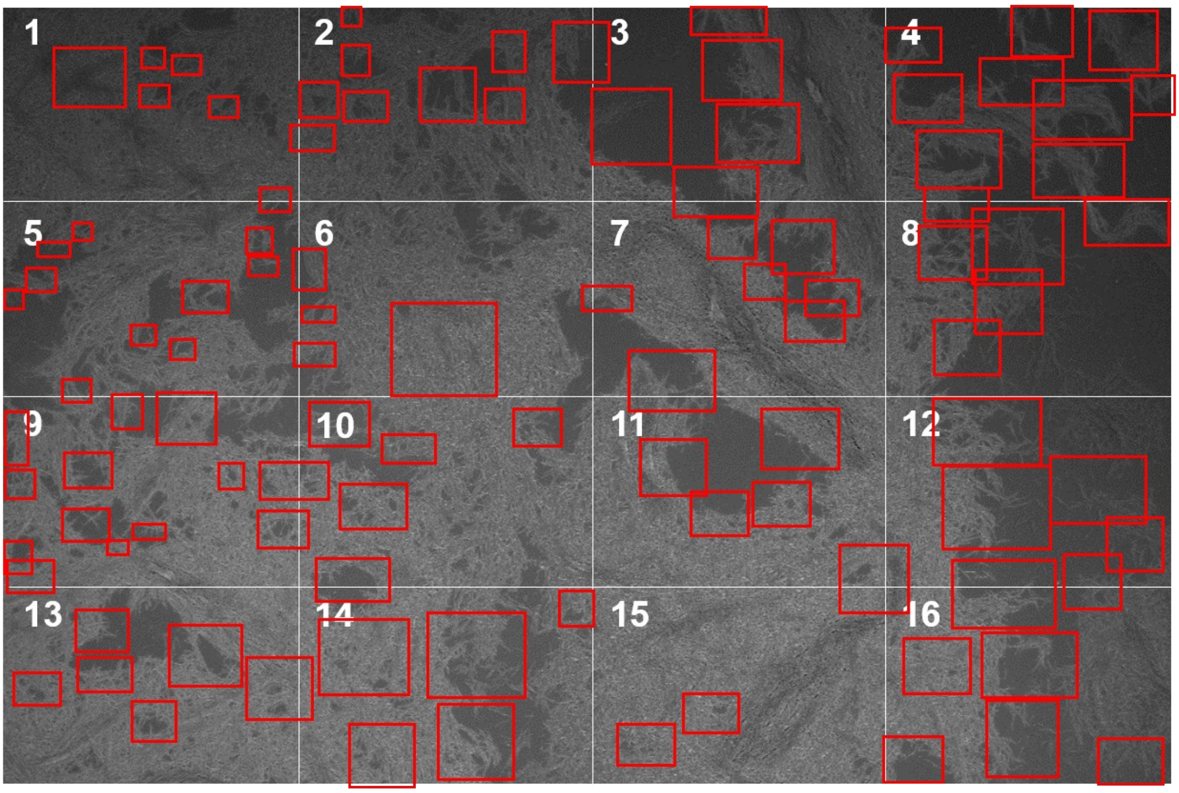

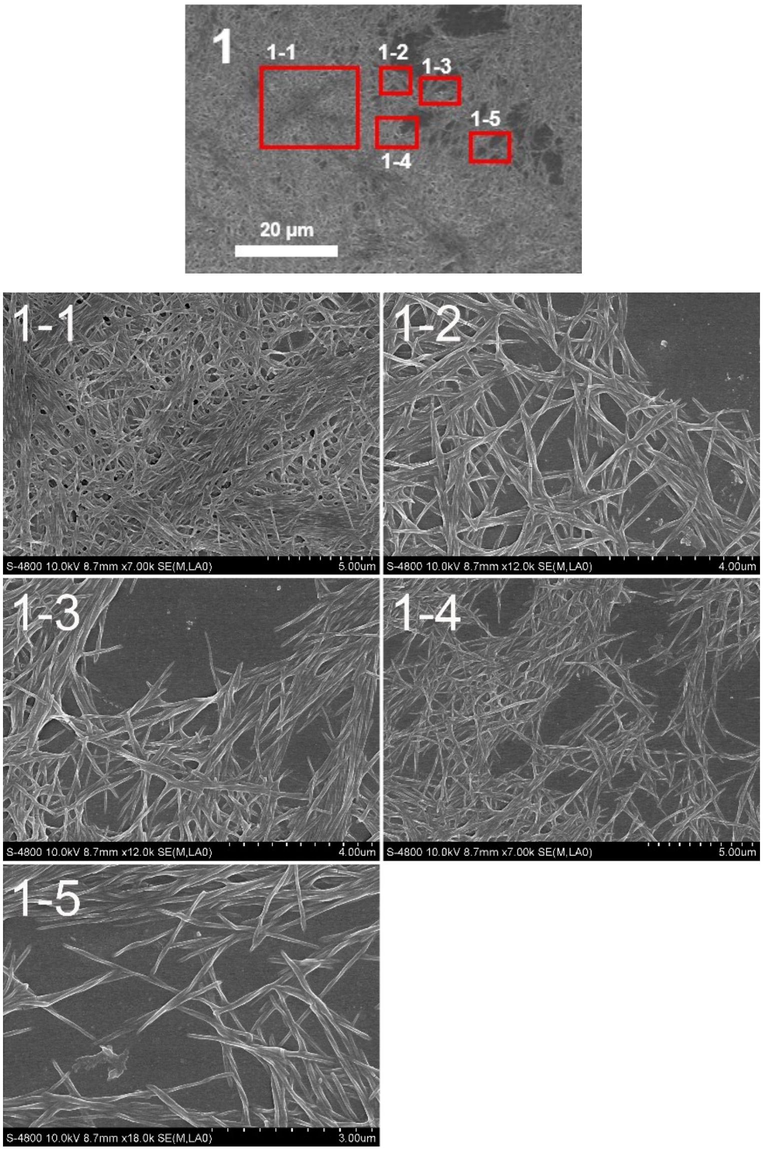
1
1-1
1-2
1-3
1-4
1-5
20 μm
S-4800 10.0kV 8.7mm x7.00k SE(M,LA0) 5.00um
S-4800 10.0kV 8.7mm x12.0k SE(M,LA0) 4.00um
S-4800 10.0kV 8.7mm x12.0k SE(M,LA0) 4.00um
S-4800 10.0kV 8.7mm x7.00k SE(M,LA0) 5.00um
S-4800 10.0kV 8.7mm x18.0k SE(M,LA0) 3.00um

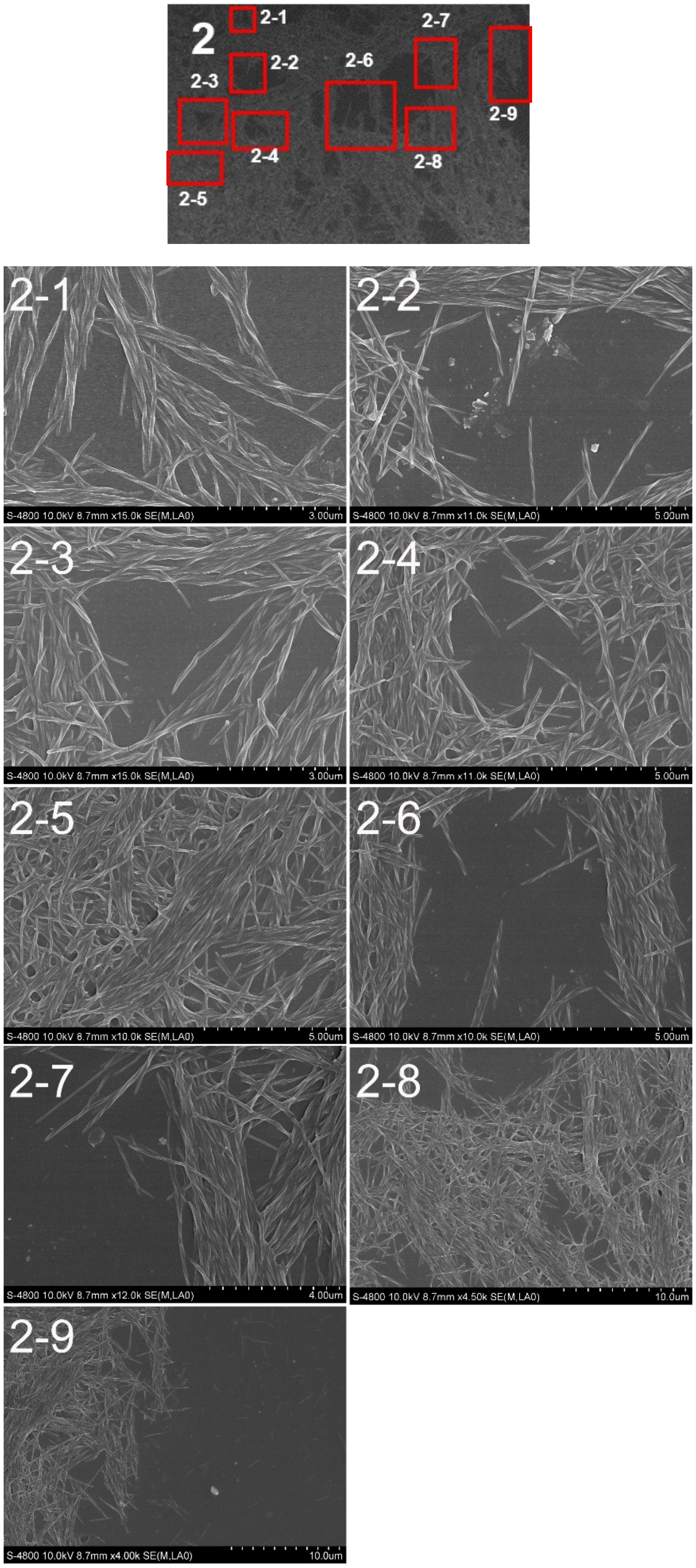
2
2-1
2-2
2-3
2-4
2-5
2-6
2-7
2-8
2-9
2-1
S-4800 10.0kV 8.7mm x15.0k SE(M,LA0)
3.00um
2-2
S-4800 10.0kV 8.7mm x11.0k SE(M,LA0)
5.00um
2-3
S-4800 10.0kV 8.7mm x15.0k SE(M,LA0)
3.00um
2-4
S-4800 10.0kV 8.7mm x11.0k SE(M,LA0)
5.00um
2-5
S-4800 10.0kV 8.7mm x10.0k SE(M,LA0)
5.00um
2-6
S-4800 10.0kV 8.7mm x10.0k SE(M,LA0)
5.00um
2-7
S-4800 10.0kV 8.7mm x12.0k SE(M,LA0)
4.00um
2-8
S-4800 10.0kV 8.7mm x4.50k SE(M,LA0)
10.0um
2-9
S-4800 10.0kV 8.7mm x4.00k SE(M,LA0)
10.0um

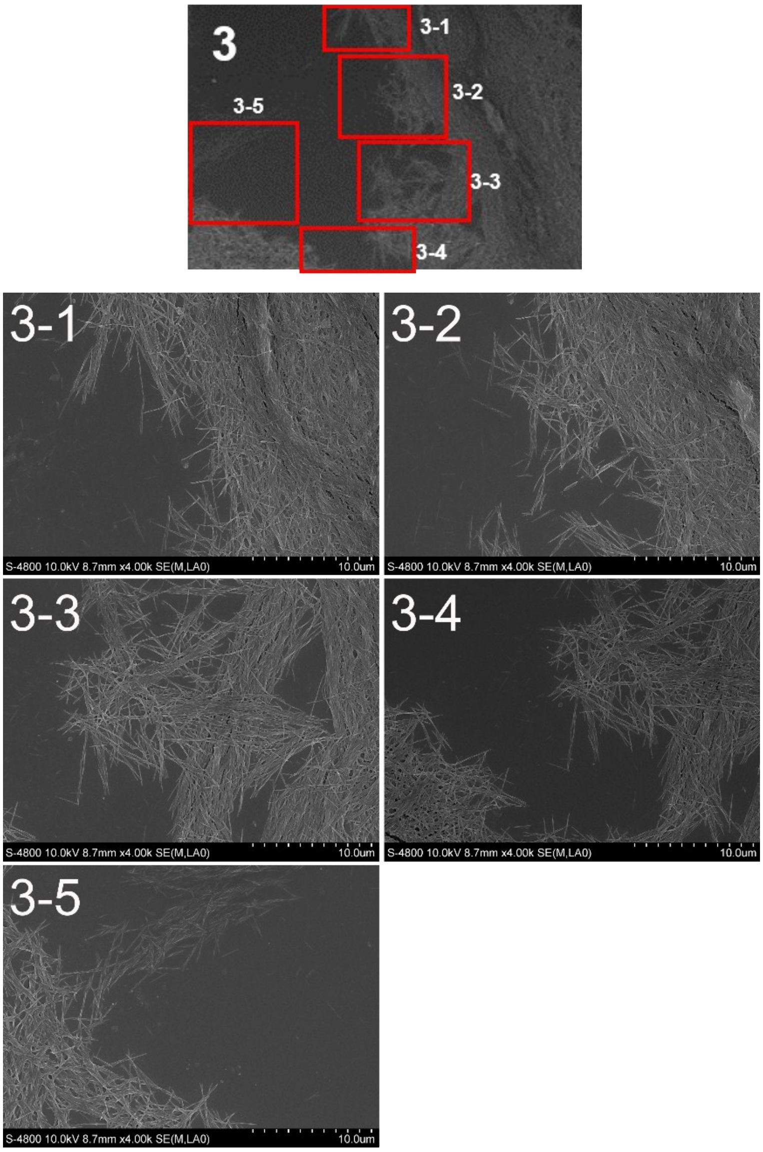
3
3-1
3-2
3-3
3-4
3-5
3-1
S-4800 10.0kV 8.7mm x4.00k SE(M,LA0)
10.0um
3-2
S-4800 10.0kV 8.7mm x4.00k SE(M,LA0)
10.0um
3-3
S-4800 10.0kV 8.7mm x4.00k SE(M,LA0)
10.0um
3-4
S-4800 10.0kV 8.7mm x4.00k SE(M,LA0)
10.0um
3-5
S-4800 10.0kV 8.7mm x4.00k SE(M,LA0)
10.0um

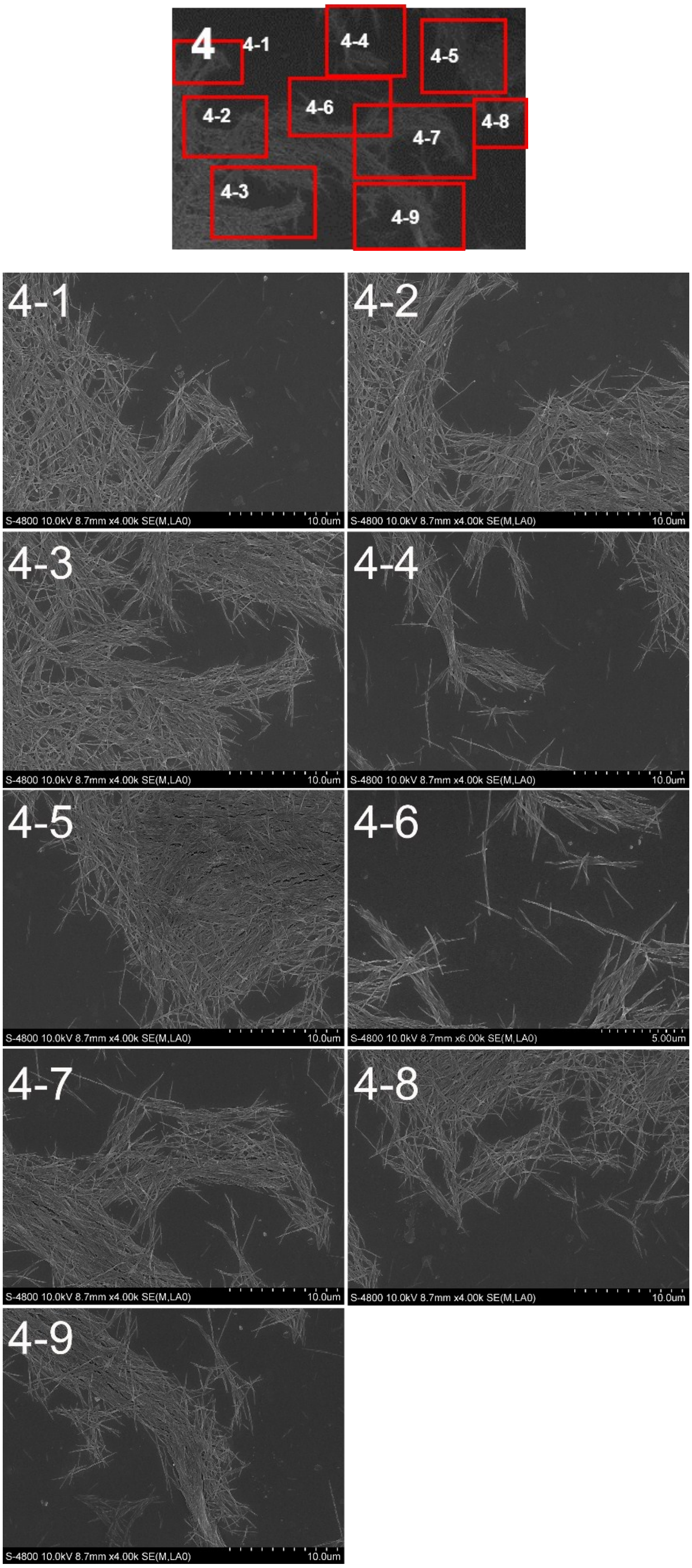
4
4-1
4-2
4-3
4-4
4-5
4-6
4-7
4-8
4-9
4-1
S-4800 10.0kV 8.7mm x4.00k SE(M,LA0) 10.0um
4-2
S-4800 10.0kV 8.7mm x4.00k SE(M,LA0) 10.0um
4-3
S-4800 10.0kV 8.7mm x4.00k SE(M,LA0) 10.0um
4-4
S-4800 10.0kV 8.7mm x4.00k SE(M,LA0) 10.0um
4-5
S-4800 10.0kV 8.7mm x4.00k SE(M,LA0) 10.0um
4-6
S-4800 10.0kV 8.7mm x6.00k SE(M,LA0) 5.00um
4-7
S-4800 10.0kV 8.7mm x4.00k SE(M,LA0) 10.0um
4-8
S-4800 10.0kV 8.7mm x4.00k SE(M,LA0) 10.0um
4-9
S-4800 10.0kV 8.7mm x4.00k SE(M,LA0) 10.0um

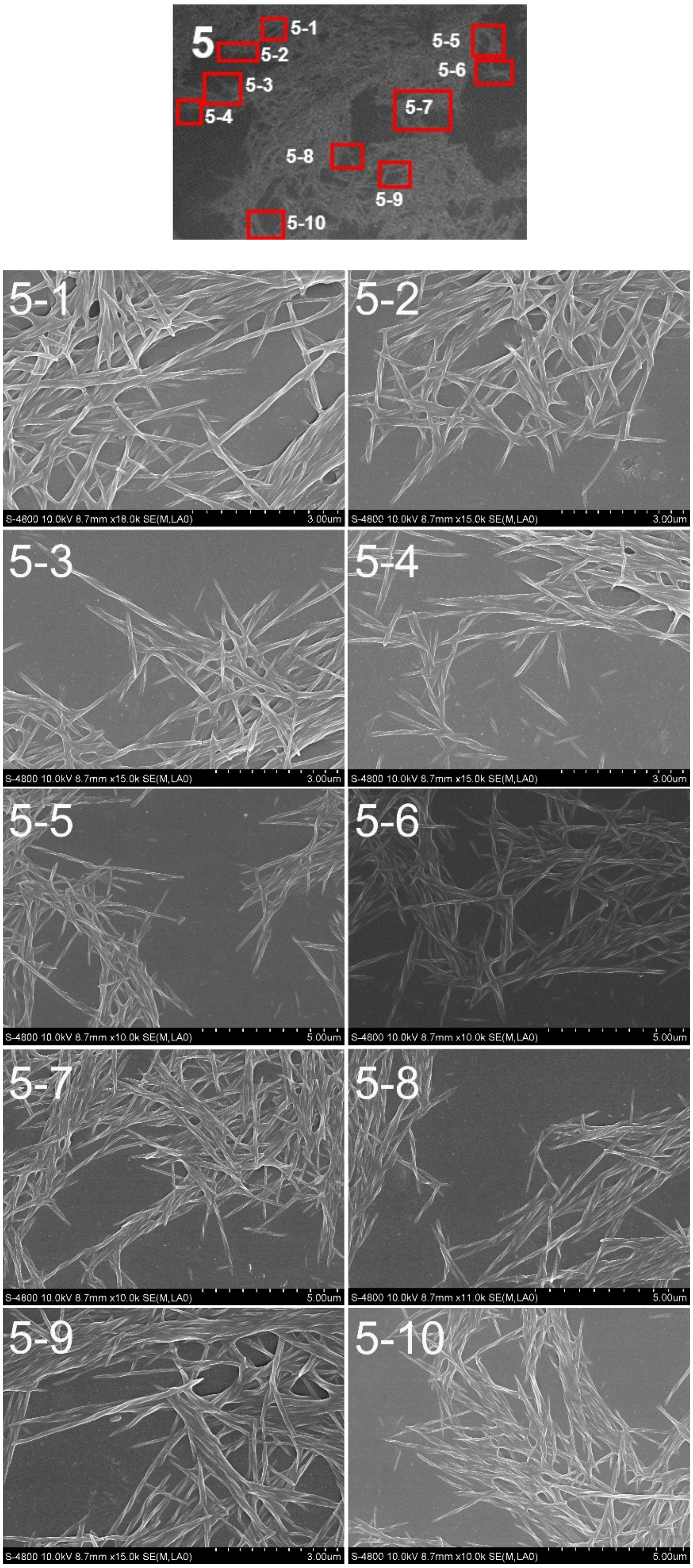
5
5-1
5-2
5-3
5-4
5-5
5-6
5-7
5-8
5-9
5-10
5-1
S-4800 10.0kV 8.7mm x18.0k SE(M,LA0) 3.00um
5-2
S-4800 10.0kV 8.7mm x15.0k SE(M,LA0) 3.00um
5-3
S-4800 10.0kV 8.7mm x15.0k SE(M,LA0) 3.00um
5-4
S-4800 10.0kV 8.7mm x15.0k SE(M,LA0) 3.00um
5-5
S-4800 10.0kV 8.7mm x10.0k SE(M,LA0) 5.00um
5-6
S-4800 10.0kV 8.7mm x10.0k SE(M,LA0) 5.00um
5-7
S-4800 10.0kV 8.7mm x10.0k SE(M,LA0) 5.00um
5-8
S-4800 10.0kV 8.7mm x11.0k SE(M,LA0) 5.00um
5-9
S-4800 10.0kV 8.7mm x15.0k SE(M,LA0) 3.00um
5-10
S-4800 10.0kV 8.7mm x10.0k SE(M,LA0) 5.00um

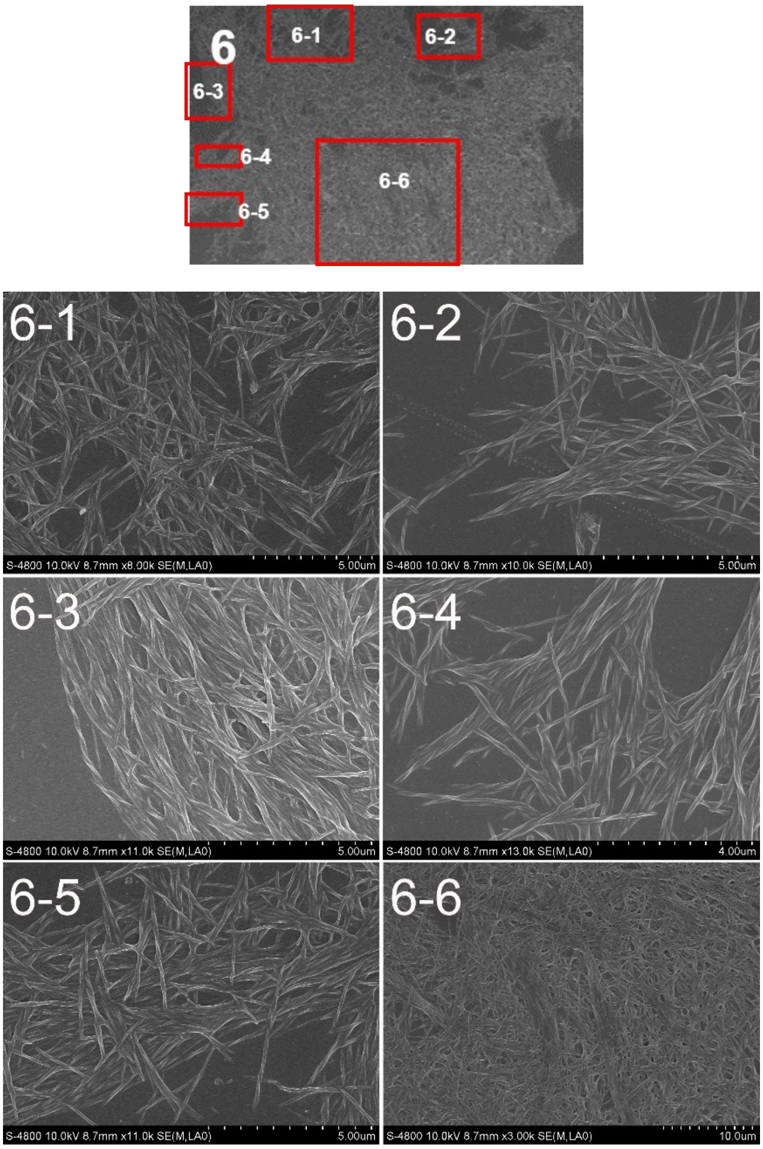
6
6-1
6-2
6-3
6-4
6-5
6-6
6-1
S-4800 10.0kV 8.7mm x8.00k SE(M,LA0)
5.00um
6-2
S-4800 10.0kV 8.7mm x10.0k SE(M,LA0)
5.00um
6-3
S-4800 10.0kV 8.7mm x11.0k SE(M,LA0)
5.00um
6-4
S-4800 10.0kV 8.7mm x13.0k SE(M,LA0)
4.00um
6-5
S-4800 10.0kV 8.7mm x11.0k SE(M,LA0)
5.00um
6-6
S-4800 10.0kV 8.7mm x3.00k SE(M,LA0)
10.0um

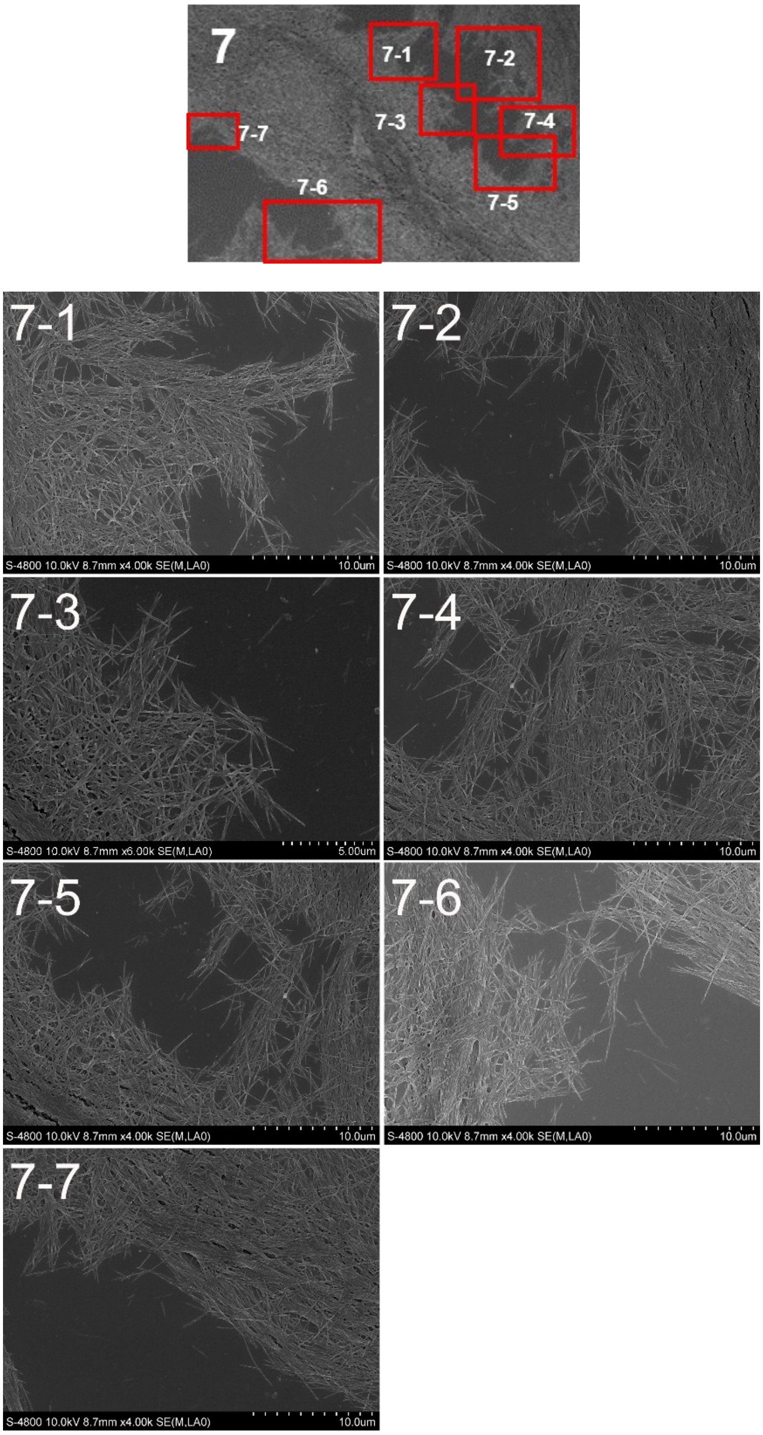
7
7-1
7-2
7-3
7-4
7-5
7-6
7-7
7-1
S-4800 10.0kV 8.7mm x4.00k SE(M,LA0) 10.0um
7-2
S-4800 10.0kV 8.7mm x4.00k SE(M,LA0) 10.0um
7-3
S-4800 10.0kV 8.7mm x6.00k SE(M,LA0) 5.00um
7-4
S-4800 10.0kV 8.7mm x4.00k SE(M,LA0) 10.0um
7-5
S-4800 10.0kV 8.7mm x4.00k SE(M,LA0) 10.0um
7-6
S-4800 10.0kV 8.7mm x4.00k SE(M,LA0) 10.0um
7-7
S-4800 10.0kV 8.7mm x4.00k SE(M,LA0) 10.0um

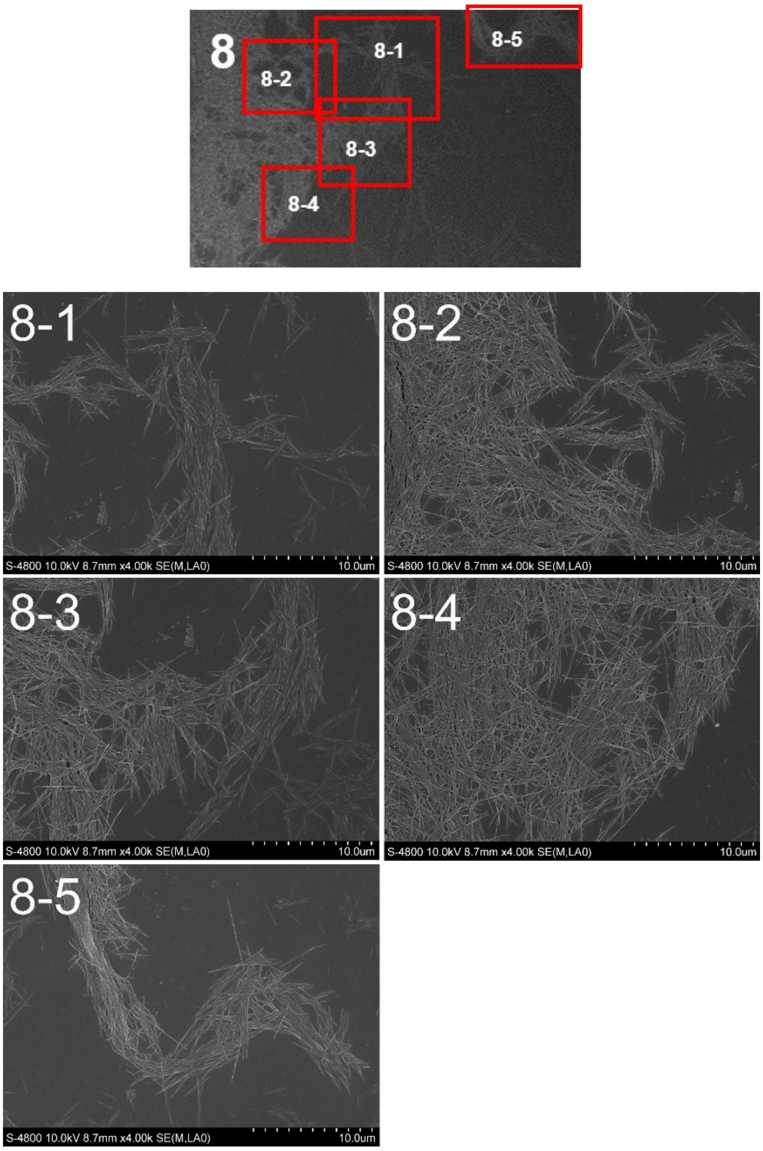

8
8-1
8-2
8-3
8-4
8-5
8-1
S-4800 10.0kV 8.7mm x4.00k SE(M,LA0)
10.0um
8-2
S-4800 10.0kV 8.7mm x4.00k SE(M,LA0)
10.0um
8-3
S-4800 10.0kV 8.7mm x4.00k SE(M,LA0)
10.0um
8-4
S-4800 10.0kV 8.7mm x4.00k SE(M,LA0)
10.0um
8-5
S-4800 10.0kV 8.7mm x4.00k SE(M,LA0)
10.0um

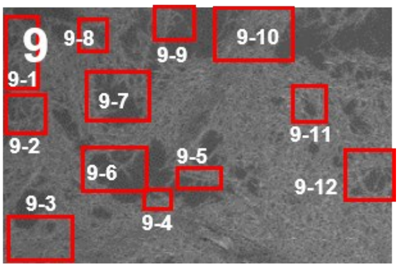
9
9-1
9-2
9-3
9-4
9-5
9-6
9-7
9-8
9-9
9-10
9-11
9-12

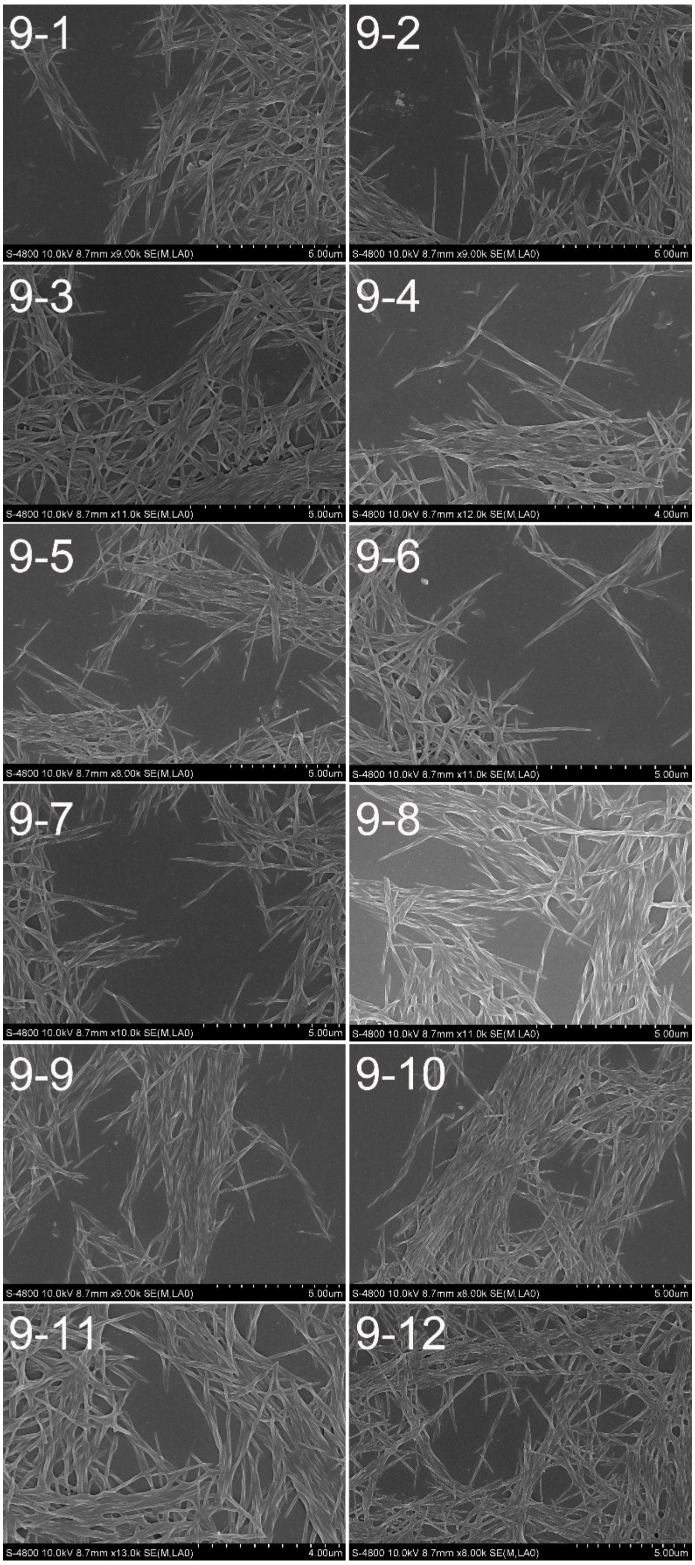
9-1
S-4800 10.0kV 8.7mm x9.00k SE(M,LA0) 5.00um
9-2
S-4800 10.0kV 8.7mm x9.00k SE(M,LA0) 5.00um
9-3
S-4800 10.0kV 8.7mm x11.0k SE(M,LA0) 5.00um
9-4
S-4800 10.0kV 8.7mm x12.0k SE(M,LA0) 4.00um
9-5
S-4800 10.0kV 8.7mm x8.00k SE(M,LA0) 5.00um
9-6
S-4800 10.0kV 8.7mm x11.0k SE(M,LA0) 5.00um
9-7
S-4800 10.0kV 8.7mm x10.0k SE(M,LA0) 5.00um
9-8
S-4800 10.0kV 8.7mm x11.0k SE(M,LA0) 5.00um
9-9
S-4800 10.0kV 8.7mm x9.00k SE(M,LA0) 5.00um
9-10
S-4800 10.0kV 8.7mm x8.00k SE(M,LA0) 5.00um
9-11
S-4800 10.0kV 8.7mm x13.0k SE(M,LA0) 4.00um
9-12
S-4800 10.0kV 8.7mm x8.00k SE(M,LA0) 5.00um

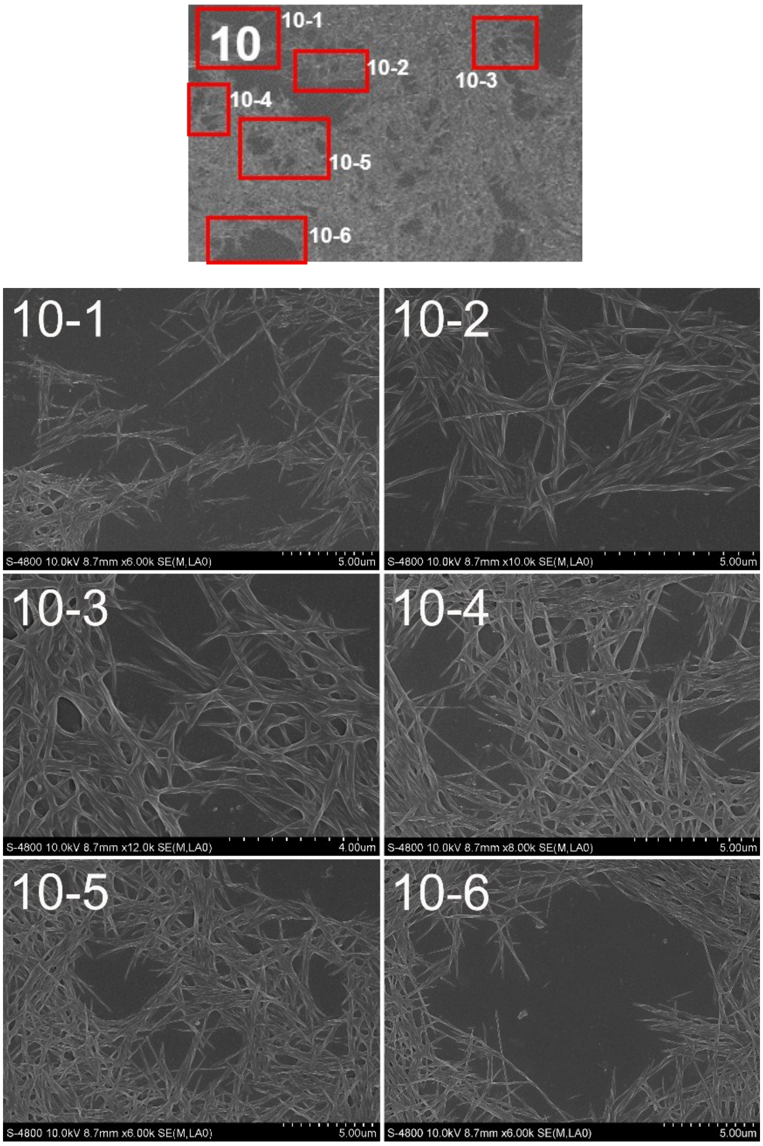
10
10-1
10-2
10-3
10-4
10-5
10-6
10-1
S-4800 10.0kV 8.7mm x6.00k SE(M,LA0)
5.00um
10-2
S-4800 10.0kV 8.7mm x10.0k SE(M,LA0)
5.00um
10-3
S-4800 10.0kV 8.7mm x12.0k SE(M,LA0)
4.00um
10-4
S-4800 10.0kV 8.7mm x8.00k SE(M,LA0)
5.00um
10-5
S-4800 10.0kV 8.7mm x6.00k SE(M,LA0)
5.00um
10-6
S-4800 10.0kV 8.7mm x6.00k SE(M,LA0)
5.00um

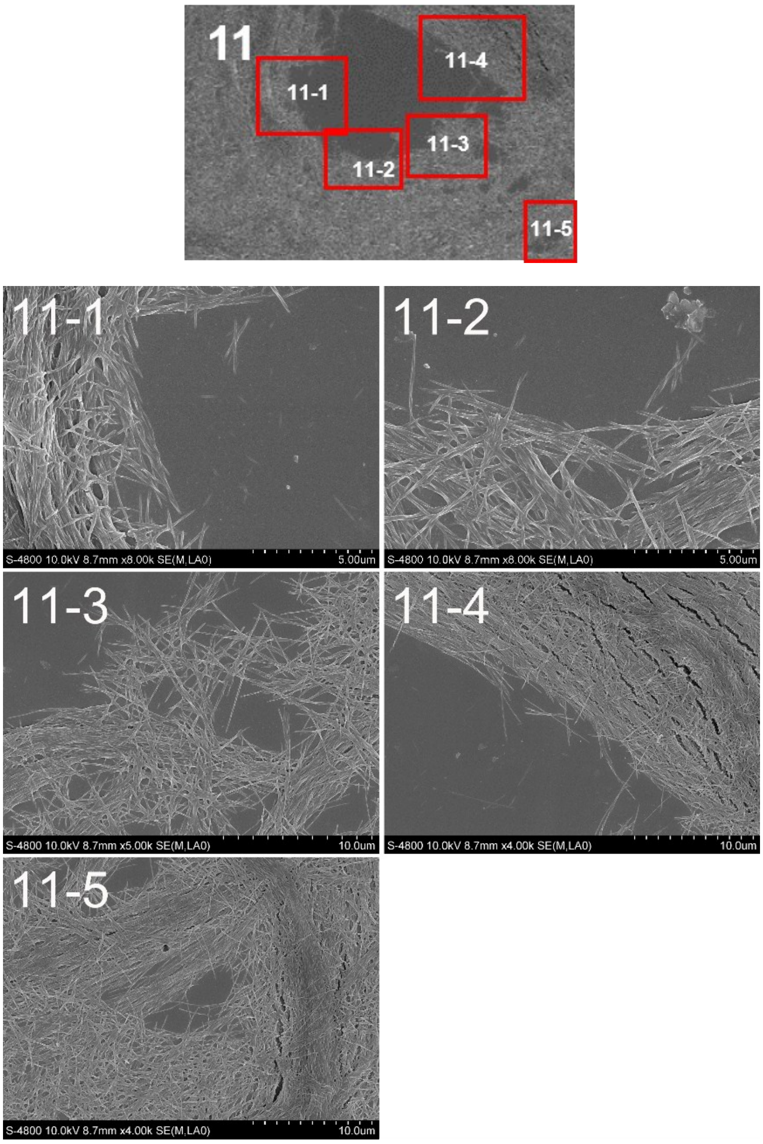
11
11-1
11-2
11-3
11-4
11-5
11-1
S-4800 10.0kV 8.7mm x8.00k SE(M,LA0)
5.00um
11-2
S-4800 10.0kV 8.7mm x8.00k SE(M,LA0)
5.00um
11-3
S-4800 10.0kV 8.7mm x5.00k SE(M,LA0)
10.0um
11-4
S-4800 10.0kV 8.7mm x4.00k SE(M,LA0)
10.0um
11-5
S-4800 10.0kV 8.7mm x4.00k SE(M,LA0)
10.0um

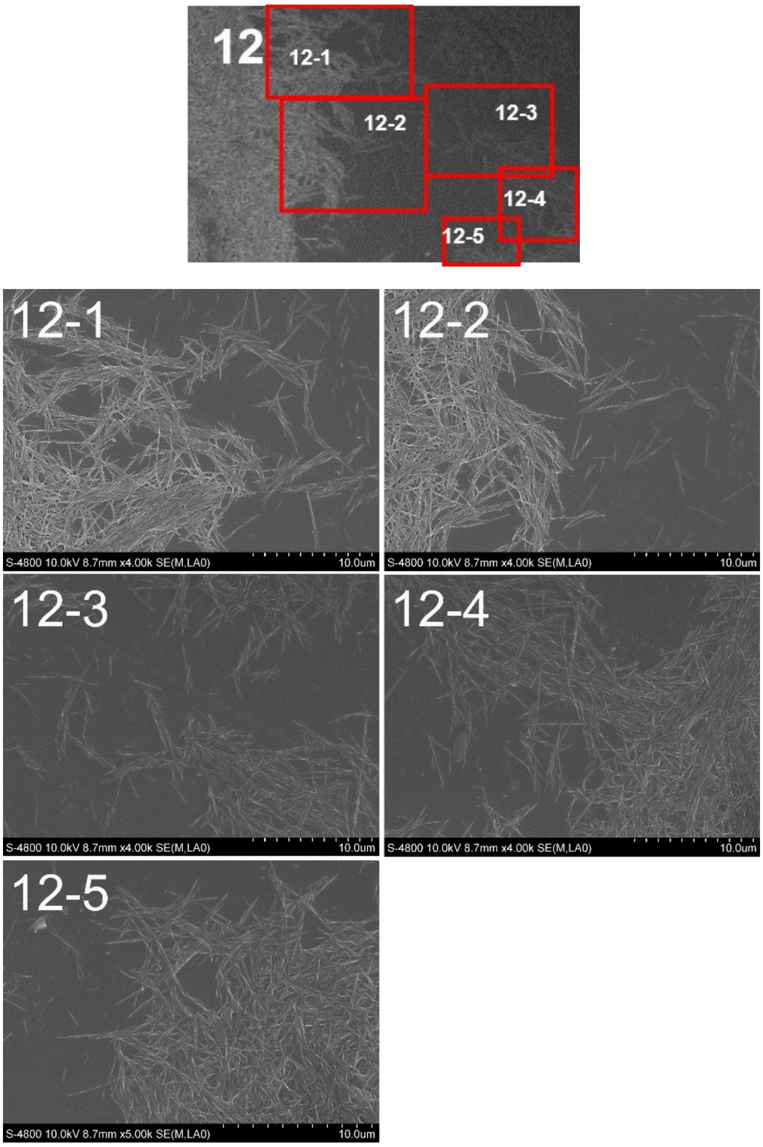
12
12-1
12-2
12-3
12-4
12-5
12-1
S-4800 10.0kV 8.7mm x4.00k SE(M,LA0)
10.0um
12-2
S-4800 10.0kV 8.7mm x4.00k SE(M,LA0)
10.0um
12-3
S-4800 10.0kV 8.7mm x4.00k SE(M,LA0)
10.0um
12-4
S-4800 10.0kV 8.7mm x4.00k SE(M,LA0)
10.0um
12-5
S-4800 10.0kV 8.7mm x5.00k SE(M,LA0)
10.0um

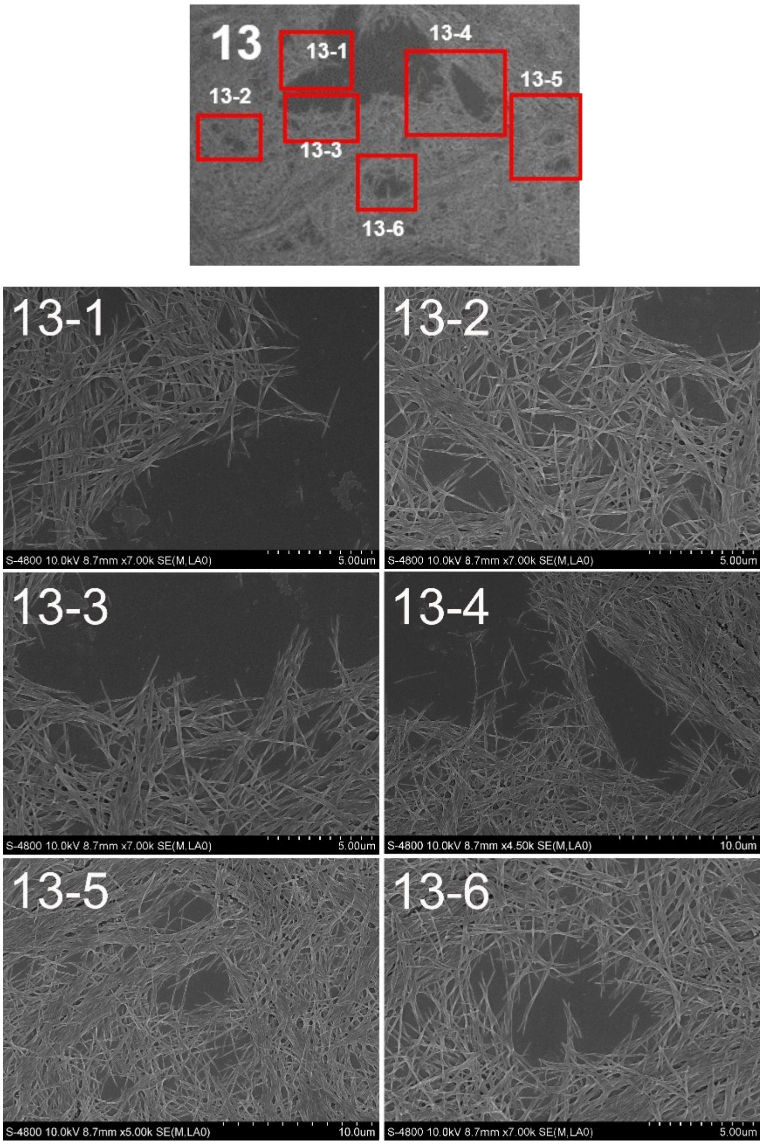
13
13-1
13-2
13-3
13-4
13-5
13-6
13-1
S-4800 10.0kV 8.7mm x7.00k SE(M,LA0)
5.00um
13-2
S-4800 10.0kV 8.7mm x7.00k SE(M,LA0)
5.00um
13-3
S-4800 10.0kV 8.7mm x7.00k SE(M,LA0)
5.00um
13-4
S-4800 10.0kV 8.7mm x4.50k SE(M,LA0)
10.0um
13-5
S-4800 10.0kV 8.7mm x5.00k SE(M,LA0)
10.0um
13-6
S-4800 10.0kV 8.7mm x7.00k SE(M,LA0)
5.00um

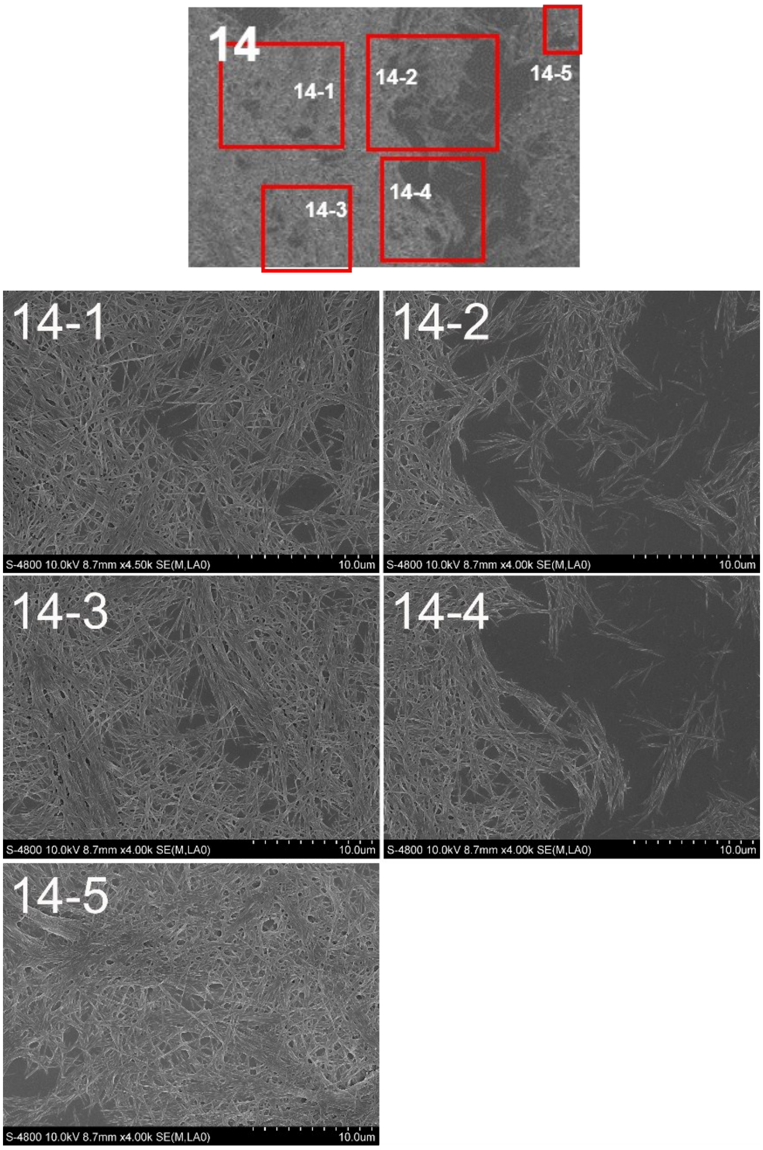
14
14-1
14-2
14-3
14-4
14-5
14-1
S-4800 10.0kV 8.7mm x4.50k SE(M,LA0)
10.0um
14-2
S-4800 10.0kV 8.7mm x4.00k SE(M,LA0)
10.0um
14-3
S-4800 10.0kV 8.7mm x4.00k SE(M,LA0)
10.0um
14-4
S-4800 10.0kV 8.7mm x4.00k SE(M,LA0)
10.0um
14-5
S-4800 10.0kV 8.7mm x4.00k SE(M,LA0)
10.0um

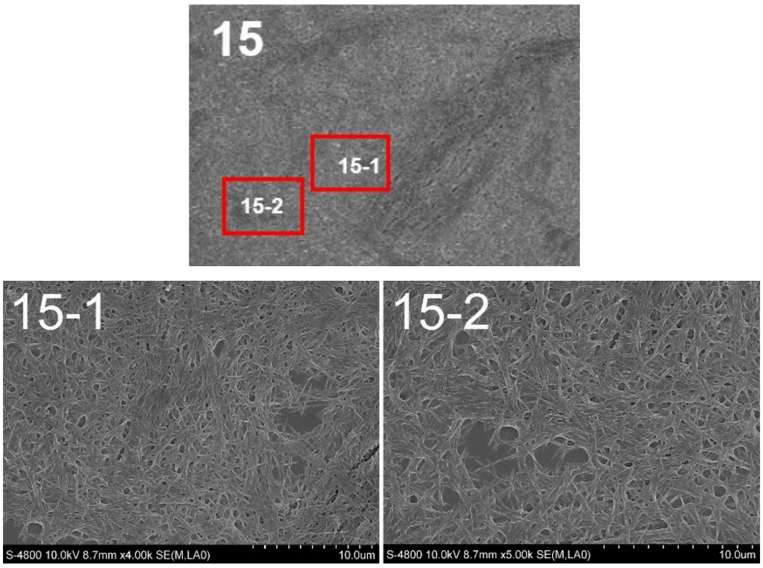
15
15-1
15-2
15-1
S-4800 10.0kV 8.7mm x4.00k SE(M,LA0)
10.0um
15-2
S-4800 10.0kV 8.7mm x5.00k SE(M,LA0)
10.0um

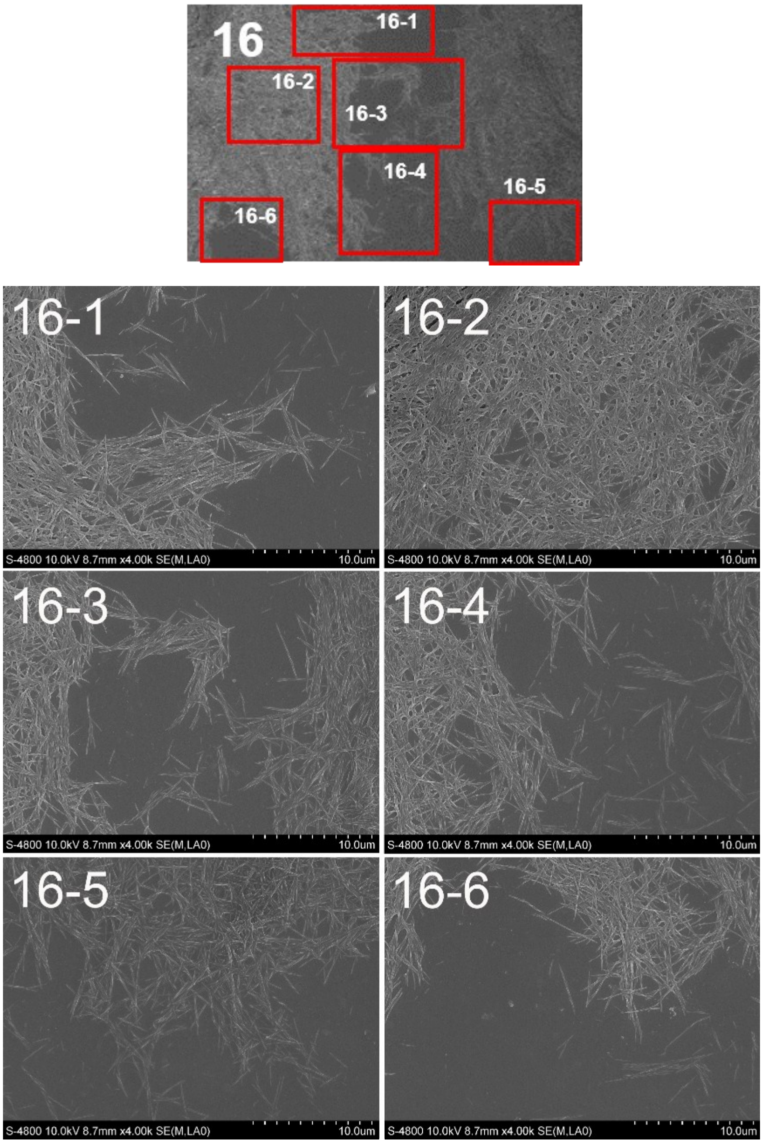
16
16-1
16-2
16-3
16-4
16-5
16-6
16-1
S-4800 10.0kV 8.7mm x4.00k SE(M,LA0)
10.0um
16-2
S-4800 10.0kV 8.7mm x4.00k SE(M,LA0)
10.0um
16-3
S-4800 10.0kV 8.7mm x4.00k SE(M,LA0)
10.0um
16-4
S-4800 10.0kV 8.7mm x4.00k SE(M,LA0)
10.0um
16-5
S-4800 10.0kV 8.7mm x4.00k SE(M,LA0)
10.0um
16-6
S-4800 10.0kV 8.7mm x4.00k SE(M,LA0)
10.0um
